# Rare Carbon-Bridged Citrinin Dimers from the Starfish-Derived Symbiotic Fungus *Penicillium* sp. GGF16-1-2

**DOI:** 10.3390/md20070443

**Published:** 2022-07-06

**Authors:** Hao Fan, Zhi-Mian Shi, Yan-Hu Lei, Mei-Xia Si-Tu, Feng-Guo Zhou, Chan Feng, Xia Wei, Xue-Hua Shao, Yang Chen, Cui-Xian Zhang

**Affiliations:** 1 School of Pharmaceutical Sciences, Guangzhou University of Chinese Medicine, Guangzhou 510006, China; 20212110040@stu.gzucm.edu.cn (H.F.); 20193102022@stu.gzucm.edu.cn (Z.-M.S.); 20201110612@stu.gzucm.edu.cn (Y.-H.L.); 20201120645@stu.gzucm.edu.cn (M.-X.S.-T.); 20201110534@stu.gzucm.edu.cn (F.-G.Z.); 18826275939@163.com (C.F.); 20193102023@stu.gzucm.edu.cn (X.W.); 2 Institute of Fruit Tree Research, Guangdong Academy of Agricultural Sciences/Key Laboratory of South Subtropical Fruit Biology and Genetic Resource Utilization (MOA)/Guangdong Province Key Laboratory of Tropical and Subtropical Fruit Tree Research, Guangzhou 510640, China; shaoxuehua@gdaas.cn

**Keywords:** starfish-derived fungus, *Penicillium* sp., citrinin dimers, antifungal activities, cytotoxic activities, protein-protein interaction network

## Abstract

Four novel, rare carbon-bridged citrinin dimers, namely dicitrinones G–J (**1**–**4**), and five known analogs (**5**–**9**) were isolated from the starfish-derived fungus *Penicillium* sp. GGF 16-1-2. Their structures were elucidated by extensive spectroscopic analysis and quantum chemical calculations. Compounds **1**–**9** exhibited strong antifungal activities against *Colletotrichum gloeosporioides* with LD_50_ values from 0.61 μg/mL to 16.14 μg/mL. Meanwhile, all compounds were evaluated for their cytotoxic activities against human pancreatic cancer BXPC-3 and PANC-1 cell lines; as a result, compound **1** showed more significant cytotoxicities than the positive control against both cell lines. In addition, based on the analyses of the protein-protein interaction (PPI) network and Western blot, **1** could induce apoptosis by activating caspase 3 proteins (CASP3).

## 1. Introduction

Dimeric natural products are a group of structurally diverse, biologically active, and biosynthetically complex metabolites. Among them, the methylene-bridged ones are a small but unique class that tend to be formed through the dimerization of two monomeric units with a methylene [1]. To date, approximately 131 natural dimers with a methylene linker were reported, including dimeric xanthones, dimeric steroids, and dimeric bioflavonoids [2,3]. Citrinin dimers are formed by the reaction of two citrinins or its analogues, including Diels-Alder-type dimers, 7, 7′ carbon-bridged citrinin dimers, and others. Among them, only seven 7, 7′ carbon-bridged-type dimers have been reported [4,5,6]. As is known to us, citrinin was one of the well-known mycotoxins [7], while citrinin dimers showed significant bioactivities compared to citrinin monomer derivatives due to their special carbon bridged skeleton, such as anti-fungi, cytotoxicity, and enzyme inhibitory activities [8,9,10].

**Figure 1:**
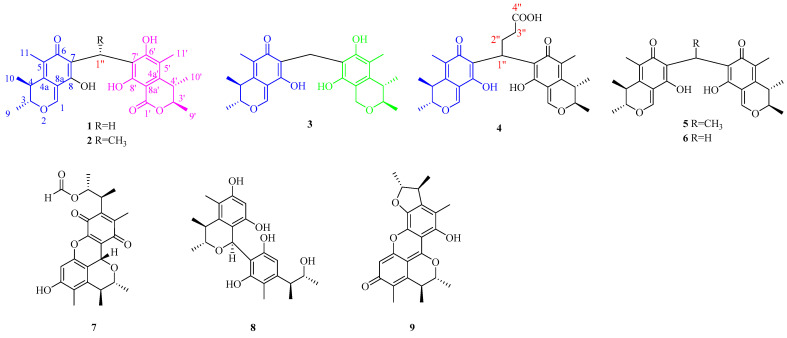
The structures of compounds **1**–**9** isolated from *Penicillium* sp. GGF 16-1-2.

As part of our continuing exploration of the novel and bioactive secondary metabolites from marine microorganisms [11,12,13], a chemical investigation of starfish-derived fungal *Penicillium* sp. GGF 16-1-2 led to the isolation and identification of four rare carbon-bridged citrinin dimers, dicitrinones G–J (**1**–**4**), known analogs Dicitrinone B (**5**) [14], Dictrinone C (**6**) [14], and known citrinin Diels-Alder-type dimers Citrinin H1 (**7**) [15,16], Penidicitrinin B (**8**) [17], and Penicitrinone A (**9**) [18,19,20] (Figure 1). We further studied their antifungal bioactivities against *Colletotrichum gloeosporioides* and antitumor activities against human pancreatic cancer cells BXPC-3 and PANC-1. The mechanism of cytotoxic activities was proposed via a protein-protein interaction network and Western blot.

## 2. Results

### 2.1. Structural Identification of New Compounds

Dicitrinone G (**1**) was isolated as orange-yellow amorphous powder with a molecular formula of C_25_H_28_O_7_, determined by its HR-ESI-MS *m*/*z* 441.1906 [M + H]^+^ (calculated: 441.1913), indicating 12 degrees of unsaturation. The absorption wavelengths in the UV spectrum peaked at 202 nm, 280 nm, and 320 nm. The IR spectra suggested hydroxyl (3280 cm^−1^), carbonyl (1639 cm^−1^), and benzene ring (1511 cm^−1^ and 1477 cm^−1^) groups. ^1^H and ^13^C NMR spectra showed one conjugated ketone carbonyl group (*δ*_C_ 187.3, C), one ester carbonyl group (*δ*_C_ 168.8, s), one tri-substituted double bond [*δ*_H_ 8.09 (s, 1H) and *δ*_C_ 107.3 (C), 157.7 (CH)], two tetra-substituted double bonds [*δ*_C_ 113.0 (C), 126.6 (C), 135.3 (C), 161.9 (C)], one fully substituted benzene ring [*δ*_C_ 98.0 (C), 111.7 (C), 115.7 (C), 140.7 (C), 157.7 (C), 161.9 (C)], two oxygenated methines [*δ*_H_ 4.71 (dq, 6.6, 13.4 1H), 4.74 (dq, 6.6, 13.4 1H) and *δ*_C_ 80.1 (CH), 80.1 (CH)], six methyl signals [*δ*_H_ 1.07 (d, 6.6, 3H), 1.17 (d, 6.6, 3H), 1.20 (d, 7.2, 3H), 1.21 (d, 7.2, 3H), 1.90 (s, 3H), 2.04 (s, 3H) and *δ*_C_ 10.0 (CH_3_), 10.3 (CH_3_), 17.6 (CH_3_), 18.4 (CH_3_), 19.5 (CH_3_), 19.6 (CH_3_)], only one methylene signal [*δ*_H_ 3.61 (d, 8.6, 1H), 3.67 (d, 8.6, 1H) and *δ*_C_ 17.2 (CH_2_)], and one active proton signal (*δ*_H_ 12.39). (Supplementary Materials Figures S4 and S5).

**Figure 2:**
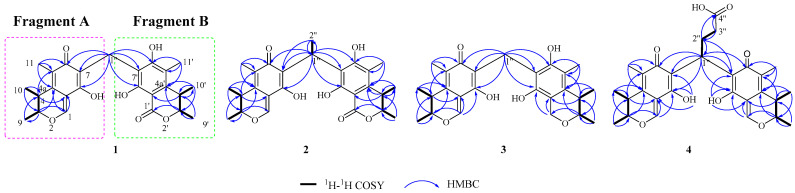
Correlation diagram of main ^1^H-^1^H COSY and HMBC of compounds **1**–**4**.

HSQC assigned attribution to their NMR data (Table 1). Careful analysis of the NMR data of compound **1** revealed the typical signals to citrinin [21,22], which suggested a similar fragment of citrinin in the structure (fragment A). Further ^1^H-^1^H COSY correlation information between H-10 (*δ*_H_ 1.20, d, 7.2)/H-4 (*δ*_H_ 3.03, dq, 7.2, 13.4)/H-3 (*δ*_H_ 4.74, dq, 6.6, 13.4)/H-9 (*δ*_H_ 1.17, d, 6.6) and HMBC correlations from H_3_-11 (*δ*_H_ 1.90, C) to C-5 (*δ*_C_ 126.6, C), C-6 (*δ*_C_ 187.3, C), and C-4a (*δ*_C_ 135.5, C); H-4 (*δ*_H_ 3.03, dq, 7.2, 13.4) to C-4a (*δ*_C_ 135.5, C), C-5 (*δ*_C_ 126.6, C), and C-8a (*δ*_C_ 107.3, C); H-1 (*δ*_H_ 8.09, s) to C-8a (*δ*_C_ 107.3, C) and C-4a (*δ*_C_ 135.5, C); H-3 (*δ*_H_ 4.74, dq, 6.6, 13.4) to C-4a (*δ*_C_ 135.5, C) and C-1 (*δ*_C_ 157.7, CH) confirmed the presence of fragment A. The remaining NMR signals indicated the existence of the fragment of dihydrocitrinone (fragment B) [23]. The ^1^H-^1^H COSY correlation information between H-10′ (*δ*_H_ 1.21, d, 7.2)/H-4′ (*δ*_H_ 3.08, dq, 7.2, 13.4)/H-3′ (*δ*_H_ 4.71, dq, 6.6, 13.4)/H-9′ (*δ*_H_ 1.07, d, 6.6), together with the HMBC correlations from H_3_-11′ (*δ*_H_ 2.04, s) to C-5′ (*δ*_C_ 115.7, C), C-6′ (*δ*_C_ 161.9, C), and C-4a′ (*δ*_C_ 140.7, C); H-4′ (*δ*_H_ 3.08, dq, 7.2, 13.4) to C-4a′ (*δ*_C_ 140.7, C), C-5′ (*δ*_C_ 115.7, C), and C-8a′ (*δ*_C_ 98.0, C) confirmed the presence of fragment B. The HMBC correlations from H_a_-1″ (*δ*_H_ 3.61, d, 8.6) and H_b_-1″ (*δ*_H_ 3.67, d, 8.6) to C-7 (*δ*_C_ 113.0, C), C-6 (*δ*_C_ 187.3, C), C-8 (*δ*_C_ 161.9, C), C-7′ (*δ*_C_ 111.7, C), C-6′ (*δ*_C_ 161.9, C), and C-8′ (*δ*_C_ 157.7, C) (Supplementary Materials Figure S9). established the planar structure of **1** by linking the above two fragments via C-1″ (Figure 2).

**Figure 3:**
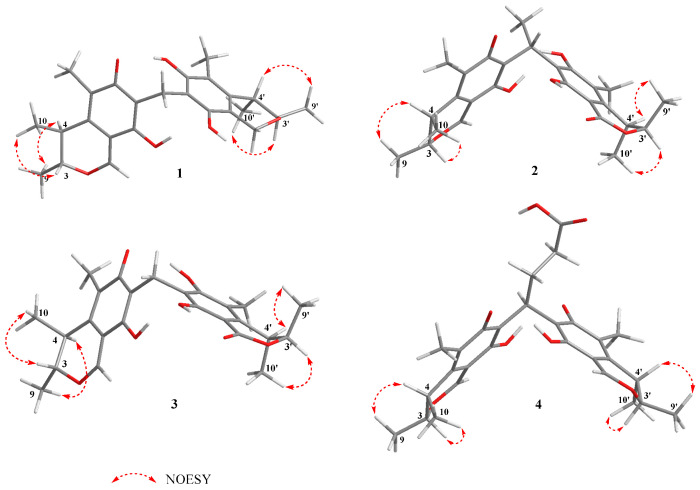
Key NOE correlations of **1**–**4**.

**Figure 4:**
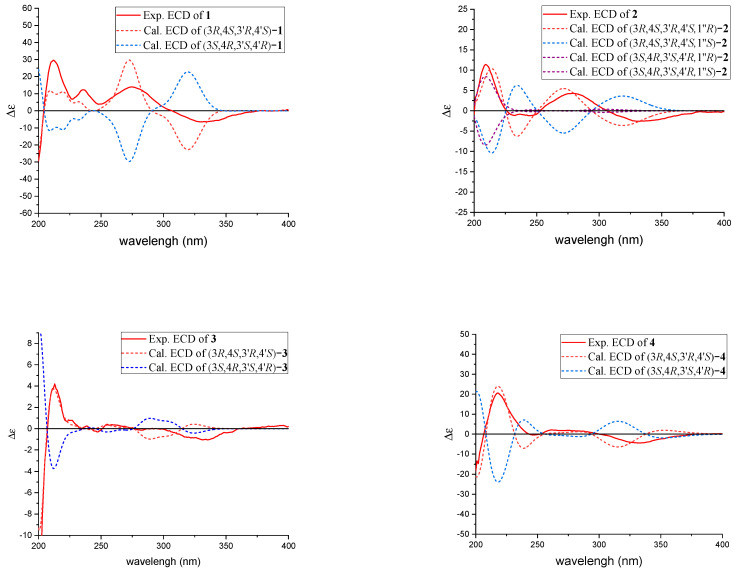
Experimental and calculated ECD spectra of **1**–**4**.

Since **1** has two stereoclusters separated by two six-membered rings joined by a methylene group, the relative configuration of each stereocluster was addressed independently. The NOESY correlation of H-4 and H_3_-9 indicated these protons were cofacial and situated in the α-orientation randomly, and the NOESY correlation of H-3 and H_3_-10 suggested that H-3 and H_3_-10 were *β*-oriented in fragment A (Supplementary Materials Figure S10). Meanwhile, the NOESY correlation of H-4′ and H_3_-9′ suggested that H-4′ and H_3_-9′ were in the *β*-orientation, while the H-3′ and H_3_-10′ were in the α-orientation in fragment B (Figure 3). Compound **1** represents a rare carbon skeleton because of the two citrinin analogues decarboxylates connected through a unique carbon-bridging center. Biosynthetically, this rare skeleton is proposed to originate from a polyketide pathway [24] and the absolute configurations of C-3, C-4, C-3′, and C-4′ were established as 3*R*, 4*S*, 3′*R*, 4′*S* [14,18,25]. To verify the absolute configurations of **1**, the quantum chemical ECD calculation was performed. By comparing the predicted ECD curves and the experimental curves, the absolute configuration of C-3, C-4, C-3′, and C-4′ of **1** was unambiguously assigned as 3*R*, 4*S*, 3′*R*, 4′*S* (Figure 4).

Dicitrinone H (**2**) was isolated as orange-yellow amorphous powder with a molecular formula of C_26_H_30_O_7_, determined by its HR-ESI-MS *m*/*z* 455.2062 [M + H]^+^ (calculated: 455.2070), indicating 12 degrees of unsaturation. NMR data of compounds **2** and **1** are extremely similar (see Table 1), indicating the same type of fragment. Compared with **1**, **2** showed an additional methyl signal [*δ*_H_ 1.55, d, (7.4) and *δ*_C_ 16.4 (CH_3_)] and methine signal [*δ*_H_ 4.89, q (7.5), and *δ*_C_ 24.1 (CH)], while the methylene signal [*δ*_H_ 3.62, q (8.6) and *δ*_C_ 17.2 (CH_2_)] in **1** was absent in **2**, indicating that **2** is a C-1″-CH_3_ derivative of **1** (Supplementary Materials Figures S14 and S15). The ^1^H-^1^H COSY correlation information between H-1” (*δ*_H_ 4.89, q, 7.5)/H-2” (*δ*_H_ 1.55, d, 7,4) and the HMBC correlations from H_3_-2″ (*δ*_H_ 1.55, d, 7.4) to C-1″ (*δ*_C_ 24.1, CH), C-7′ (*δ*_C_ 115.7, C) and C-7 (*δ*_C_ 117.1, C) were further confirmed with the above proposal (Figure 2).

Similarly, the relative configuration of **2** was deduced based on the NOESY correlations and the absolute configuration was established as 3*R*, 4*S*, 1″*R*, 3′*R*, 4′*S* according to biological pathways [24] and the quantum chemical ECD calculation.

Dicitrinone I (**3**) was isolated as orange-yellow colloidal with a molecular formula of C_25_H_30_O_6_, determined by its HR-ESI-MS *m*/*z* 427.2114 [M + H]^+^ (calculated: 427.2121), indicating 11 degrees of unsaturation. Comprehensive analyses of the 1D NMR (Table 1) spectra of **3** with those of **1** suggested **3** was highly similar to **1**. The major differences between them were the appearance of an oxygenated methylene signal [*δ*_H_ 4.49 (s) and *δ*_C_ 59.0 (CH_2_)] in **3** instead of a carbonyl carbon signal [*δ*_C_ 168.8 (C)] in **1**, suggesting that **3** is a hydrogenated analogue of **1**. The observed HMBC correlations from H_2_-1′ (*δ*_H_ 4.49, s) to C-8a′ (*δ*_C_ 114.6, C), C-8′ (*δ*_C_ 147.9, C), C-4a′ (*δ*_C_ 135.1, C), and C-3′ (*δ*_C_ 73.3, CH) were further confirmed based on the above assumption.

Similar to **1**, based on the NOESY correlations, the relative configuration of **3** was deduced and based on the subsequent analyses of the biological pathways [24] and the quantum chemical ECD calculation, the absolute configuration of **3** was established as 3*R*, 4*S*, 3′*R*, 4′*S*.

Dicitrinone J (**4**) was isolated as a yellow-brown amorphous powder with a molecular formula of C_28_H_32_O_8_, determined by its HR-ESI-MS *m*/*z* 497.2165 [M + H]^+^ (calculated: 497.2175), indicating 13 degrees of unsaturation. The ^1^H and ^13^C NMR spectra (recorded in DMSO-*d*_6_) revealed a mixture of two atropisomers at a ratio of approximately 1:1 according to the ^1^H NMR integration. Comprehensive analyses of the NMR spectra of **4** (Table 1) indicated that **4** was a citrinin dimer with a symmetric structure (Figure 2). The ^1^H-^1^H COSY correlation information between H-1” (*δ*_H_ 4.29/4.32, t, 7.2), H-2” (*δ*_H_ 2.36/2.36, m), and H-3” (*δ*_H_ 2.04/2.04, m) and the observed HMBC correlations from H-1″ (*δ*_H_ 4.29/4.32, t, 7.2) to C-2″ (*δ*_C_ 23.6/23.9, CH_2_) and C-3″ (*δ*_C_ 32.7, CH_2_), from H-2″ (*δ*_H_ 2.36/2.36, m) to C-1′′ (*δ*_C_ 30.5/30.4, CH) and C-4′′ (*δ*_C_ 174.0/174.1, C), from H-3″ (*δ*_H_ 2.04/2.04, m) to C-1′′ (*δ*_C_ 30.5/30.4, CH), C-2″ (*δ*_C_ 23.6/23.9, CH_2_), and C-4′′ (*δ*_C_ 174.0/174.1, C); at the same time, the observed correlations from H-1″ (*δ*_H_ 4.29/4.4.32, t, 7.2) to C-6 (*δ*_C_ 186.1/186.3, C), C-6′ (*δ*_C_ 186.6/186.6, C), C-7 (*δ*_C_ 116.1/116.2, C), C-7′ (*δ*_C_ 116.3/116.6, C), C-8 (*δ*_C_ 163.4/164.0, C), and C-8′ (*δ*_C_ 163.8/163.8, C) indicated a butyric acid chain which attached to two citrinin decarboxylates through a C-7, C-1″, and C-7′ bridge. (Figure 2). The butyric acid chain, which increased the molecular asymmetry, could hinder the free rotation of the two citrinin decarboxylate segments through the -C-7/7′-C-1″- single bond. Two atropisomers were stabilized due to restricted rotation and the intramolecular hydrogen bonds between the two segments. This observation was confirmed by the active hydrogen resonances [*δ*_H_ 13.33 (OH-8′) and 13.15 (OH-8)] in the ^1^H NMR spectrum (Supplementary Materials Figure S34). These combined analyses could explain why the two atropisomers were captured simultaneously by NMR in a 1:1 ratio.

Similar to **1**, based on the NOESY correlations, the relative configuration of **4** was deduced and based on the subsequent analyses of the biological pathways [24] and the quantum chemical ECD calculation, the absolute configuration of **4** was established as 3*R*, 4*S*, 3′*R*, 4′*S*. Compounds **5**–**9** (Figure 1) were identified as the known Dicitrinone B (**5**) [14], Dictrinone C (**6**) [14], Citrinin H1 (**7**) [15,16], Penidicitrinin B (**8**) [17], and Penicitrinone A (**9**) [18,19,20], by comparing their NMR data with that reported in the literature.

### 2.2. Evaluation of Antifungal Activity

*Colletotrichum gloeosporioides*, an important phytopathogenic fungus, mainly infects tropical fruits and results in serious anthracnose [26]. Compounds **1**–**9** were tested by mycelial growth rate assay against *Colletotrichum gloeosporioides* [27]. The results (Table 2) showed that **6** had the strongest antifungal activities against *Colletotrichum gloeosporioides* with LC_50_ values of 0.61 μg/mL. According to their structural characteristics, citrinin monomers and methylene bridges may be important to the antifungal activities. Their bioactivities decreased when the methylene bridge was replaced by an alkane, or the length of the alkane bridge was increased. Meanwhile, if citrinin was oxidized or reduced, its antifungal activities would be weakened.

### 2.3. Cytotoxic Assays

As there is no clinically effective drug for pancreatic cancer [28], we selected Doxorubicin hydrochloride as the positive control for the cytotoxic activity test. Based on the cytotoxicity assay [29] (Table 3), **1** was more significantly cytotoxic against human BXPC-3 cell lines than the Doxorubicin hydrochloride and similar results were obtained against human PANC-1 cell lines with the positive control.

**Figure 5:**
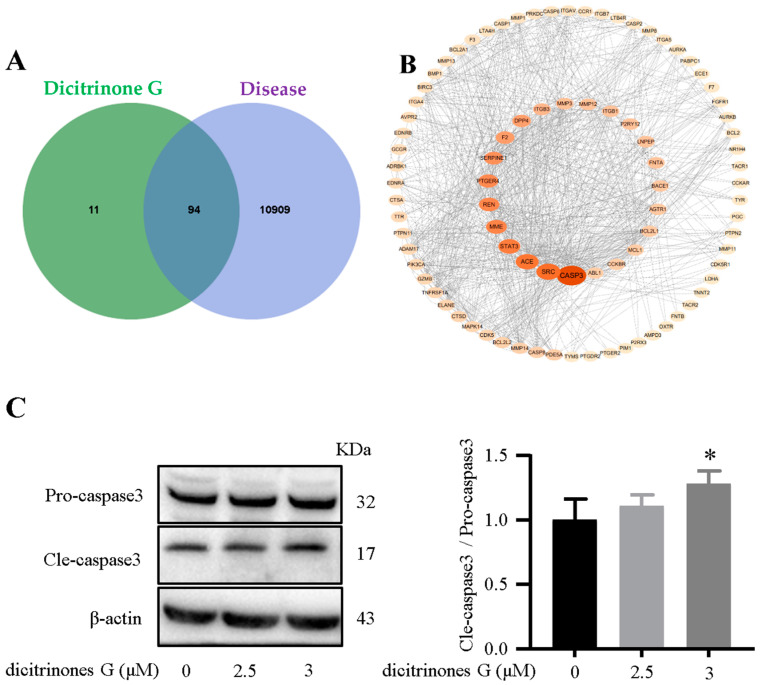
(**A**) The Venn diagram of **1** and the disease to obtain overlapping genes. (**B**) The PPI network of **1** and the disease. (**C**) The effects of **1** on caspase 3 proteins in BXPC-3 cells were determined by western blot analysis. All data are expressed as means ± SD. * *p* < 0.05 vs. the control group.

To explore the possible mechanism of the cytotoxic activity of **1**, the top 105 potential mechanism genes for **1** have been predicted with the Swiss Target Prediction database [30]; genes related to pancreatic cancer were selected from the GeneCards database to construct the receptor database and 94 overlapping genes were obtained from the Venn diagram. A protein-protein interaction (PPI) network was established by the STRING 11.5 database [31] and Cytoscape 3.9.0 software to screen the critical targets and the size and the color of the symbols represented the degree scores in the network analysis [32]. The analysis results of the PPI indicated that CASP3 has the most degree scores, which means CASP3 could be the essential protein for **1** (Figure 5 A,B). We verified it via a Western blot experiment in vitro and found that **1** could affect CASP3 expression (Figure 5C), which reportedly plays a crucial role in the cell apoptosis pathway [33,34,35]. Therefore, we hypothesized that **1** might promote BXPC-3 apoptosis by affecting the activation of CASP3.

## 3. Materials and Methods

### 3.1. General Experimental Procedures

Details of the instrumentations and materials used in this work are included in the Supplementary Materials.

### 3.2. Fungal Materials, Extraction, and Fermentation

*Penicillium* sp. GGF 16-1-2 fungus was isolated from starfish in the South China Sea [36]. The strain was stored in the Laboratory of Marine Natural Medicine, School of Pharmaceutical Sciences, Guangzhou University of Chinese Medicine (No. GGF16-1-2).

The fungus *Penicillium* sp. GGF 16-1-2 was cultured under static conditions at 28 °C in 1 L Erlenmeyer flasks containing 400 mL of the culture medium comprising 10 g monosodium glutamate, 40 g maltose, 13 g yeast extract, 3 g magnesium sulfate heptahydrate, 5 g monopotassium phosphate, 5 g tryptophan, 1 L pure water, and 50 g sorbitol (in seawater). After 60 days of cultivation, 30 L of whole broth was filtered through cheesecloth to separate the supernatant from the mycelia. The former was extracted three times with EtOAc. The culture was extracted thrice with EtOAc and the pooled organic solvent was evaporated to dryness under vacuum to afford a crude extract (111.9 g).

Soybean culture medium: 50 g (≥24 mesh) soybean grains, 33% sea salt, 85 mL pure water, pH natural. A 5.0 mL seed solution was inoculated into soybean culture medium (50 g/1 L/bottle) and a total of 10 L was cultured. The medium was placed in a room at 28 °C for 45 days. The ethyl acetate extract was soaked with ethyl acetate (500 mL ethyl acetate/bottle, 24 h/time, 3–5 times). The ethyl acetate extract was condensed under reduced pressure to yield 5.8 g of the EtOAc residue.

### 3.3. Isolation

The EtOAc soluble fraction (111.9 g) was subjected to a silica gel column chromatography (Si CC, 165 kg, 10 cm × 110 cm) and eluted with a gradient of petroleum ether-ethyl acetate (V_PE_:V_EtOAc_ = 100:0 to 0:100, *v*/*v*) to afford six fractions [Fr.1 (0.4 g), Fr.2 (4.0 g), Fr.3 (10.0 g), Fr.4 (5.9 g), Fr.5 (40.0 g), and Fr.6 (18.9 g)]. Fr.2 (4.0 g) was separated by Sephadex LH-20 gel column chromatography (Sephadex LH-20, 100 g, 3 cm × 200 cm) and eluted with methyl alcohol to yield five subfractions (Fr.2-1–Fr.2-5). Fr.2-1 (339 mg) was fractionated by SP-HPLC (Kromasil semi-preparative column, 10 mm × 250 mm, 5 µm Akzo Nobel, Sweden), eluting with MeOH:H_2_O (V_MeOH_:V_H__2O_ = 80:20, 2.0 mL/min) to yield compounds **5** (*t*_R_ = 49.14 min, 15.0 mg) and **1** (*t*_R_ = 48.9 min, 45.8 mg). Fr.2-4 (1.0 g) was purified by HPLC (flow rate: 1.5 mL/min) with V_MeOH_: V_H__2O_ = 80:20 as the mobile phase, yielding compound **2** (*t*_R_ = 64.7 min, 35.5 mg). Fr.2-5 (2.5 g) was purified by HPLC (flow rate: 1.5 mL/min) with V_MeOH_:V_H__2O_ = 80:20 as the mobile phase, yielding compound **6** (*t*_R_ = 66.1 min, 31.9 mg).

Fr.3 (10.0 g) was recrystallized to obtain a red solid by MeOH, namely compound **7** (33 mg). The MeOH soluble fraction was separated by Sephadex LH-20 gel column chromatography (Sephadex LH-20, 100 g, 3 cm × 200 cm) and eluted with MeOH to yield five subfractions (Fr.3-1–Fr.3-5). Fr.3-3 (1.7 g) was purified by HPLC (flow rate: 1.5 mL/min) with MeOH:H_2_O = 80:20 as the mobile phase to afford 15 peaks. Peak 4 (*t*_R_ =13.2 min, 52.1 mg) was further purified on a SP-HPLC (V_MeOH_:V_H__2O_ = 75:25, 4.0 mL/min) to obtain **3** (*t*_R_ = 47.3 min, 21.7 mg); peak 5 (*t*_R_ =14.1 min, 32.7 mg) was purified again on a SP-HPLC (V_MeOH_:V_H__2O_ = 75:25, 2.0 mL/min) to yield **8** (*t*_R_ = 38.4 min, 3.3 mg) and, similarly, compound **4** (*t*_R_ = 38.4 min, 55.7 mg) was isolated from peak 7 (*t*_R_ =18.3 min, 111.8 mg).

The ethyl acetate extract of soybean extract (5.8 g) was separated by ODS (400 g, 40–60, Φ = 5.5 cm, l = 43/62 cm, column volume 800 mL), eluting with V_MeOH_:V_H__2O_ (40:60, 50:50, 60:40, 70:30, 80:20, 90:10, 100:0) to afford eight fractions (Fr.1-Fr.8). Fr.6 (160.3 mg) was purified by HPLC (PFP chromatographic column: ACE 10 C-18-PFP, 250 mm × 10 mm, 4.0 mL/min) with V_MeOH_:V_H__2O_ = 70:30 as the mobile phase, yielding compound **9** (*t*_R_ = 24.4 min; 33.0 mg).

### 3.4. Structural Characterizations of the New Compounds ***1***–***4***

Dicitrinone G (**1**): orange-yellow amorphous powder (MeOH), [α${]}_{D}^{20}$ −35.2 (*c* 0.10, MeOH); UV (MeOH) *λ*_max_ (log *ε*): 202 (3.74), 280 (3.07), 320 (3.02) nm; IR (neat) *ν*_max_: 3280, 1639, 1511, 1477cm^−1^; HRESIMS *m*/*z*: 441.1906 [M + H]^+^ (calcd for C_25_H_29_O_7_, 441.1913 [M + H]^+^); ^1^H NMR (400 MHz) and ^13^C NMR (100 MHz) data in DMSO-*d*_6_, see Table 1.

Dicitrtinone H (**2**): orange-yellow amorphous powder (MeOH), [α${]}_{D}^{20}$ −82.7 (*c* 0.10, MeOH); UV (MeOH) λ_max_ (log *ε*): 202 (2.42) nm, 280 (4.04) nm, 320 (4.26) nm; IR (neat) *ν*_max_: 3259 cm^−1^, 1718 cm^−1^, 1646 cm^−1^, 1509 cm^−1^, 1452 cm^−1^; HRESIMS *m*/*z*: 455.2062 [M + H]^+^ (calcd for C_26_H_31_O_7_,455.2070 [M + H]^+^); ^1^H NMR (400 MHz) and ^13^C NMR (100 MHz) data in DMSO-*d*_6_, see Table 1.

Dicitrinone I (**3**): orange-yellow colloidal (MeOH), [α${]}_{D}^{20}$ −50.9 (*c* 0.13, MeOH); UV (MeOH) λ_max_ (log *ε*): 280 (3.11) nm, 343 (4.44) nm; IR (neat) *ν*_max_: 3288 cm^−1^, 1644 cm^−1^, 1544 cm^−1^; HRESIMS *m*/*z*: 427.2114 [M + H]^+^ (calcd for C_25_H_31_O_6_, 427.2121 [M + H]^+^); ^1^H NMR (400 MHz) and ^13^C NMR (100 MHz) data in DMSO-*d*_6_, see Table 1.

Dicitrinone J (**4**): yellow-brown amorphous powder (MeOH), [α${]}_{D}^{20}$ −82.7 (*c* 0.10, MeOH); UV (MeOH) λ_max_ (log *ε*): 202 (3.72) nm, 280 (2.63) nm, 340 (3.16) nm; IR (neat) *ν*_max_: 3403 cm^−1^, 1718 cm^−1^, 1631 cm^−1^; HRESIMS *m*/*z*: 497.2165 [M + H]^+^(calcd for C_28_H_33_O_8_, 497.2175 [M + H]^+^); ^1^H NMR (400 MHz) and ^13^C NMR (100 MHz) data in DMSO-*d*_6_, see Table 1.

### 3.5. Antifungal Activity Assay

Initial evaluations of the antifungal activity of the purified compounds were conducted against *Colletotrichum gloeosporioides* by mycelial growth rate assay. Compound solutions with different concentrations were prepared (three replicates for each concentration) and poured into petri dishes for later use. A PDA medium plate with sterile water was used as a control. The cultured pathogen cakes were taken with a sterile perforator and inoculated into the center of the PDA medium plate. The pathogen cakes were placed in a constant temperature incubator at 28 °C for 3 days. The positive control was carbendazim. The colony diameter was measured by the cross-bonded method and the inhibition rate of mycelium growth was calculated.

### 3.6. Cytotoxic Assays

The toxicity vitalities of **1**–**9** and doxorubicin hydrochloride were examined by the MTT assays. A 100 μL cell suspension in the culture medium was added into a 96-well plate with a seeding density of 7000 cells per well. The plate was incubated at 37 °C in 5% CO_2_ for 12 h. Then the medium was replaced with freshly prepared growth media containing **1**–**9** at different concentrations of 0 μM, 1 μM, 2 μM, 4 μM, 8 μM, 16 μM, 32 μM, 64 μM, and 128 μM. After 24 h of incubation, 20 μL of 5 mg/mL MTT solution was then added to each well. After 4 h, the MTT medium was removed and 200 μL DMSO was added to each well. After incubating for 10 min, the absorbance at 570 nm was determined with a plate reader.

### 3.7. Quantum Chemical Calculations

The random conformational searchs of **1**–**4** were performed by the SYBYL X 2.1.1 program using a MMFF94s molecular force field, with an energy cutoff of 10 kcal mol^−1^ to the global minima, which afforded 8, 11, 12, and 19 conformers, respectively. All the obtained conformers were subsequently optimized by using Gaussion09 software at B3LYP/6-31+G(d) level in the gas phase, which afforded 6, 4, 4, and 11 stable conformers, respectively. These optimized stable conformers were next subjected for further ECD calculations at the B3LYP/6-31+G(d) level in methanol. The overall ECD spectrums of **1**–**4** were weighted by Boltzmann distribution and subsequently compared with the experimental ones, respectively. The ECD spectra were produced by SpecDis 1.70.1 software [11,12,13,37,38,39,40,41].

### 3.8. Targets Prediction

The SMILES format files of compound **1** were uploaded to the Swiss Target Prediction database (http://www.swisstargetprediction.ch/; accessed on 15 May 2022) to predict the target’s information. The GeneCards database (https://www.genecards.org; accessed on 17 May 2022) was used to predict potential targets for pancreatic cancer. The STRING database (https://string-db.org/cgi/input.pl; accessed on 18 May 2022) analyzed the common gene symbols to construct a PPI network. The network analysis was visualized by Cytoscape 3.9.0 software based on the score of the protein interaction.

### 3.9. Western Blot Assays

BXPC-3 cells were incubated at 37 °C under 5% CO_2_ atmosphere. For quantitative Western blot analysis, 70–80% confluent cells were seeded at 2 × 10^5^/per well onto 6-well plates for 12 h, followed by stimulation with 2.5 μM, 3.0 μM **1** for 24 h. The cultured cells were first washed twice with precooled PBS, followed by the addition of a RIPA lysis buffer combined with a mixture of proteases or phosphatase inhibitors to lyse the total protein, and then the protein concentration was quantified by a BCA protein assay kit (Solarbio, Beijing, China) according to the manufacturer’s instructions. Equal amounts of protein extract were separated on a 12% SDS-PAGE gel and electrotransferred to 0.22 mm PVDF membranes using a Bio-Rad wet transfer tank. After blocking with 5% nonfat milk at room temperature for 2 h, membranes were incubated with the specific antibodies targeting caspase 3 (wanlei, 1:1000) and β-actin (Affinity, 1:1000) at 4 °C overnight. After incubating with the appropriate horseradish peroxidase (HRP)-conjugated secondary antibodies (CST), protein bands were detected using an enhanced chemiluminescence kit (Millipore, WBLUR0500) and imaged. Band intensities were quantified using Image J software.

### 3.10. Statistical Analysis

Statistical analysis was performed using GraphPad Prism and SPSS 22.0 software. Data are expressed as the mean ± standard deviation (SD) and are representative of at least three experiments. *p* < 0.05 was considered to indicate a statistically significant difference.

## 4. Conclusions

Four novel, rare carbon-bridged citrinin dimers were discovered from the starfish-derived symbiotic fungus *Penicillium* sp. GGF16-1-2. Compounds **1**–**6** were typical 7, 7′carbon-bridged citrinin dimers, likely formed by decarboxylation, dehydration, reduction, and condensation of short-chain fatty acids of carboxyl carbon on two citrinin units C-7 [10,14,20,21]. Because of structural specificity, **6** showed strong antifungal activity against *Colletotrichum gloeosporioides*, with LC_50_ values of 0.61 μg/mL. Meanwhile, **1** showed significant cytotoxicity against human pancreatic cancer cell lines BXPC-3 and PANC-1. We further attempted to propose the possible mechanism by network pharmacology and Western blot and it showed that **1** might promote BXPC-3 apoptosis by affecting the activation of CASP3.

## Figures and Tables

**Table 1:** ^1^H NMR data (400 MHz) and ^13^C NMR data (100 MHz) of **1**–**4** (*δ* in ppm, *J* in Hz) in DMSO-*d*_6_.

No.	1		2		3		4	
	*δ*_H_, Mult, *J*	*δ*_C_, Mult	*δ*_H_, Mult, *J*	*δ*_C_, Mult	*δ*_H_, Mult, *J*	*δ*_C_, Mult	*δ*_H_, Mult, *J*	*δ*_C_, Mult
1	8.09, s	157.7, CH	8.07, s	158.7, CH	8.00, s	157.7, CH	8.00, s/8.01, s	158.0/158.2, CH
3	4.74, dq (6.6, 13.4)	80.1, CH	4.69, dq (6.4, 12.9)	79.8, CH	4.70, dq (6.6, 13.1)	79.9, CH	4.72, m/4.72, m	79.8/80.0, CH
4	3.03, dq (7.2, 13.4)	33.6, CH	2.99, dq (7.1, 12.9)	33.5, CH	2.99, dq (7.2, 13.1)	33.5, CH	2.99, m/2.99, m	33.5, CH
4a		135.5, C		136.2, C		136.0, C		136.2/136.3, C
5		126.6, C		126.6, C		124.4, C		124.2/124.6, C
6		187.3, C		187.8, C		184.5, C		186.1/186.3, C
7		113.0, C		117.1, C		112.8, C		116.1/116.2, C
8		161.9, C		160.6, C		161.5, C		163.4/164.0, C
8a		107.3, C		107.0, C		106.9, C		106.8/106.9, C
9	1.17, d (6.6)	19.5, CH_3_	1.22, d (6.4)	19.4, CH_3_	1.18, d (6.6)	17.6, CH_3_	1.22, d (6.6)/1.22, d (6.6)	17.5/17.5, CH_3_
10	1.20, d (7.2)	17.6, CH_3_	1.16, d (7.1)	17.6 CH_3_	1.06, d (7.2)	18.3, CH_3_	1.11, d (7.1)/1.11, d (7.1)	18.2/18.3, CH_3_
11	1.90, s	10.0, CH_3_	1.88, s	9.9, CH_3_	1.89, s	10.0, CH_3_	1.87, s/1.87, s	9.7/9.8, CH_3_
1′		168.8, C		168.9, C	4.49, s	59.0, CH_2_	8.03, s/8.04, s	158.8/159.0, CH
3′	4.71, dq (6.6, 13.4)	80.1, CH	4.72, dq (6.5, 12.9)	79.8, CH	3.77, m	73.3, CH	4.73, m/4.73, m	80.1/80.1, CH
4′	3.08, dq (7.2, 13.4)	33.6, CH	3.05, dq (7.1, 12.9)	33.5, CH	2.50, m	34.5, CH	2.99, m/2.99, m	33.5, CH
4a′		140.7, C		140.2, C		135.1, C		136.9/136.9, C
5′		115.7, C		117.1, C		114.1, C		125.0/125.3, C
6′		161.9, C		162.7, C		150.5, C		186.6/186.6, C
7′		111.7, C		115.7, C		113.8, C		116.3/116.6, C
8′		157.7, C		158.7, C		147.9, C		163.8/163.8, C
8a′		98.0, C		97.6, C		114.6, C		107.2/107.4, C
9′	1.07, d (6.6)	18.4, CH_3_	1.09, d (6.5)	18.4, CH_3_	1.08, d (6.6)	17.8, CH_3_	1.15, d (6.6)/1.15, d (6.6)	17.7/17.7, CH_3_
10′	1.21, d (7.2)	19.6, CH_3_	1.17, d (7.1)	19.6, CH_3_	1.09, d (7.2)	20.5, CH_3_	1.04, d (7.1)/1.04, d (7.1)	18.5/18.5, CH_3_
11′	2.04, s	10.3, CH_3_	2.02, s	10.4, CH_3_	2.02, s	11.0, CH_3_	1.87, s/1.87, s	10.1/10.1, CH_3_
1″	a 3.61, d (8.6) b 3.67, d (8.6)	17.2, CH_2_	4.89, q (7.5)	24.1, CH	3.57, *br* s	18.1, CH_2_	4.29, t (7.2)/4.32, t (7.2)	30.5/30.4, CH
2″			1.55, d (7.4)	16.4, CH_3_			2.36, m/2.36, m	23.6/23.9, CH_2_
3″							2.04, m/2.04, m	32.7, CH_2_
4″								174.0/174.1, C
8-OH							13.15, s	
6′-OH	12.39, s		12.90, s					
8′-OH							13.33, s	

**Table 2:** Antifungal activities of compounds **1**–**9** (LD_50_, µg/mL).

Compd.	*Colletotrichum gloeosporioides*	Compd.	*Colletotrichum gloeosporioides*
**1**	16.14	**6**	0.61
**2**	10.23	**7**	5.31
**3**	9.58	**8**	7.58
**4**	9.63	**9**	4.34
**5**	8.87	Carbendazim *	49.58

* Carbendazim serves as a positive control.

**Table 3:** Cytotoxic activities of **1**–**9** in BXPC-3 and PANC-1 cell lines (IC_50_, μM).

Compd.	BXPC-3	PANC-1
**1**	12.25 ± 2.85	24.33 ± 2.10
**2**	>50	39.54 ± 2.50
**3**	>50	>50
**4**	>50	>50
**5**	>50	>50
**6**	>50	>50
**7**	32.25 ± 3.82	49.85 ± 1.11
**8**	>50	>50
**9**	>50	>50
Doxorubicin hydrochloride *	18.24 ± 2.84	24.00 ± 3.65

* Doxorubicin hydrochloride serves as a positive control.

## Data Availability

Not applicable.

## Supplementary Materials

The following are available online at https://www.mdpi.com/article/10.3390/md20070443/s1, Figures S1–S40: the HRESIMS, IR, UV, 1D NMR, and 2D NMR spectra of compounds **1**–**4**; Figures S41–S44: Key molecular orbitals involved in important transitions regarding the ECD spectrum of the dominant conformer of **1**–**4**; Table S1–S8: Cartesian coordinate of the dominant conformer and key transitions and their related rotatory and oscillator strengths of the dominant conformer of **1**–**4** at the B3LYP/6-31+g(d) level.
