# Supplementary material for: Rare Carbon-Bridged Citrinin Dimers from the Starfish-Derived Symbiotic Fungus Penicillium sp. GGF16-1-2
Source: Mar Drugs. 2022 Jul 6;20(7):443. doi: 10.3390/md20070443 (PMC9317178; doi:10.3390/md20070443)
Supplement: Supplementary file 1 [file marinedrugs-20-00443-s001.zip › marinedrugs-1789108-supplementary.pdf]

Supporting Information

Rare Carbon-bridged Citrinin Dimers from the Starfish-derived Symbiotic Fungus *Penicillium* sp. GGF16-1-2

Hao Fan [1,†], Zhi-Mian Shi [1,†], Yan-Hu Lei [1], Mei-Xia Si-Tu [1], Feng-Guo Zhou [1], Chan Feng [1], Xia Wei [1], Xue-Hua Shao [2], Yang Chen [1,*], Cui-Xian Zhang [1,*]

List of Content

General experimental procedures.

Figure S1. HRESIMS Spectrum of compound **1**.

Figure S2. IR Spectrum of compound **1**.

Figure S3. UV Spectrum of compound **1**.

Figure S4. ^{1}H NMR (400 MHz) Spectrum of compound **1** in DMSO-d_6.

Figure S5. ^{13}C NMR (100 MHz) Spectrum of compound **1** in DMSO-d_6.

Figure S6. DEPT 135 (100 MHz) Spectrum of compound **1** in DMSO-d_6.

Figure S7. COSY Spectrum of compound **1** in DMSO-d_6.

Figure S8. HSQC Spectrum of compound **1** in DMSO-d_6.

Figure S9. HMBC Spectrum of compound **1** in DMSO-d_6.

Figure S10. NOESY Spectrum of compound **1**.

Figure S11. HRESIMS Spectrum of compound **2**.

Figure S12. IR Spectrum of compound **2**.

Figure S13. UV Spectrum of compound **2**.

Figure S14. ^{1}H NMR (400 MHz) Spectrum of compound **2** in DMSO-d_6.

Figure S15. ^{13}C NMR (100 MHz) Spectrum of compound **2** in DMSO-d_6.

Figure S16. DEPT 135 (100 MHz) Spectrum of compound **2** in DMSO-d_6.

Figure S17. COSY Spectrum of compound **2** in DMSO-d_6.

Figure S18. HSQC Spectrum of compound **2** in DMSO-d_6.

Figure S19. HMBC Spectrum of compound **2** in DMSO-d_6.

Figure S20. NOESY Spectrum of compound **2**.

Figure S21. HRESIMS Spectrum of compound **3**.

Figure S22. IR Spectrum of compound **3**.

Figure S23. UV Spectrum of compound **3**.

Figure S24. ^{1}H NMR (400 MHz) Spectrum of compound **3** in DMSO-d_6.

Figure S25. ^{13}C NMR (100 MHz) Spectrum of compound **3** in DMSO-d_6.

Figure S26. DEPT 135 (100 MHz) Spectrum of compound **3** in DMSO-d_6.

Figure S27. COSY Spectrum of compound **3**in DMSO-d_6.

Figure S28. HSQC Spectrum of compound **3** in DMSO-d_6.

Figure S29. HMBC Spectrum of compound **3** in DMSO-d_6.

Figure S30. NOESY Spectrum of compound **3** in DMSO-d_6.

Figure S31. HRESIMS Spectrum of compound **4.**

Figure S32. IR Spectrum of compound **4.**

Figure S33. UV Spectrum of compound **4.**

Figure S34. ^{1}H NMR (400 MHz) Spectrum of compound **4** in DMSO-d_6.

Figure S35. ^{13}C NMR (100 MHz) Spectrum of compound **4** in DMSO-d_6.

Figure S36. DEPT 135 (100 MHz) Spectrum of compound **4** in DMSO-d_6.

Figure S37. COSY Spectrum of compound **4** in DMSO-d_6.

Figure S38. HSQC Spectrum of compound **4** in DMSO-d_6.

Figure S39. HMBC Spectrum of compound **4** in DMSO-d_6.

Figure S40. NOESY Spectrum of compound **4** in DMSO-d_6.

Quantum chemical ECD calculations of 1−4

Table S1. Cartesian coordinate of dominant conformer of **1** (B3LYP/6-31+g(d)).

Table S2. Key transitions and their related rotatory and oscillator strengths of dominant conformer of **1** at the B3LYP/6-31+g(d) level.

Figure S41. Key molecular orbitals involved in important transitions regarding the ECD spectrum of dominant conformer of **1**.

Table S3. Cartesian coordinate of dominant conformer of **2** (B3LYP/6-31+g(d))

Table S4. Key transitions and their related rotatory and oscillator strengths of dominant conformer of **2** at the B3LYP/6-31+g(d) level.

Figure S42. Key molecular orbitals involved in important transitions regarding the ECD spectrum of dominant conformer of **2**.

Table S5. Cartesian coordinate of dominant conformer of **3** (B3LYP/6-31+g(d)).

Table S6. Key transitions and their related rotatory and oscillator strengths of dominant conformer of **3** at the B3LYP/6-31+g(d) level.

Figure S43. Key molecular orbitals involved in important transitions regarding the ECD spectrum of dominant conformer of **3**.

Table S7. Cartesian coordinate of dominant conformer of **4** (B3LYP/6-31+g(d)).

Table S8. Key transitions and their related rotatory and oscillator strengths of dominant conformer of **4** at the B3LYP/6-31+g(d) level.

Figure S44. Key molecular orbitals involved in important transitions regarding. the ECD spectrum of dominant conformer of **4**.

General experimental procedures

Silica gel (200~300 mesh, Qingdao Marine Chemical Factory), Sephadex LH-20 (GE Healthare, Sweden), ODS (ODS-A-HG, YMC, Japan), GF254 silica gel plate (Qingdao Marine Chemical Factory). Methanol, ethyl acetate, petroleum ether and dichloromethane were used for column chromatography (all analytically pure, Tianjin Damao Chemical Reagent Factory). HPLC with methanol and acetonitrile (chromatographic pure, Beijing Mairuida Technology Co., LTD.), ultrapure water. BRUKE 400MHz NMR instrument (Germany Bruker Company), MCP200 digital polarimeter (AntonPaar, Austria), Chirascan circular discoloration Spectrometer (Applied Optical Physics, UK), Triple TOFTM 5600+ mass spectrometry system (AB, USA) SCIEX), Nicolet6700-Continuμm Fourier Transform Infrared spectroscopy-Microscope (Thermo Scientific corporation of the United States), QuikSep Semi-preparative high performance liquid chromatography (HPLC) instrument (Beijing Huideyi Technology Co., LTD.), Chromatographic column: Kromasil semi-preparation column (10 mm×250 mm, 5 μm, Akzo Nobel, Sweden), Kromasil preparation column (21 mm×250 mm, 5 μm, Akzo Nobel, Sweden), YMC-Pack ODS-A semi-prepared column (10 mm×250 mm, 5 μm, YMC, Japan), PFP semi-prepared column (10 mm×250 mm, 5 μm, Guangzhou Philomen Scientific Instrument Co., LTD.). Biochemical incubator (Huangshi Hengfeng Medical Instrument Co., LTD.). Silica gel (200~300 mesh, Qingdao Marine Chemical Factory), Sephadex LH-20 (GE Healthare, Sweden), ODS (ODS-A-HG, YMC, Japan), GF254 silica gel plate (Qingdao Marine Chemical Factory). Methanol, ethyl acetate, petroleum ether and dichloromethane were used for column chromatography (all analytically pure, Tianjin Damao Chemical Reagent Factory). HPLC with methanol and acetonitrile (chromatographic pure, Beijing Mairuida Technology Co., LTD.), ultrapure water.

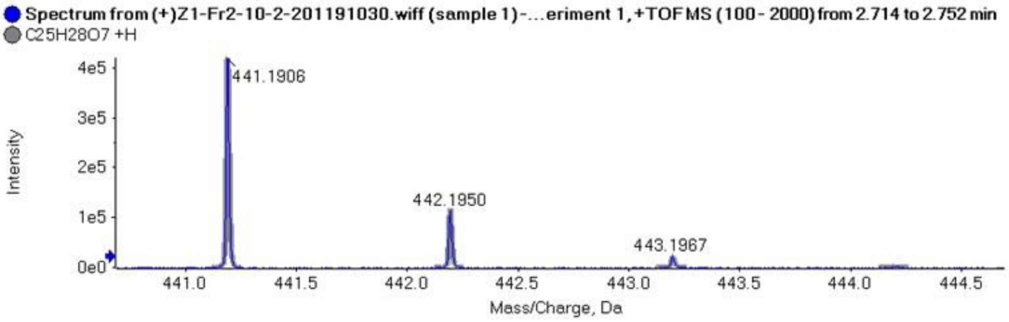

Figure S1. HRESIMS Spectrum of compound **1**.

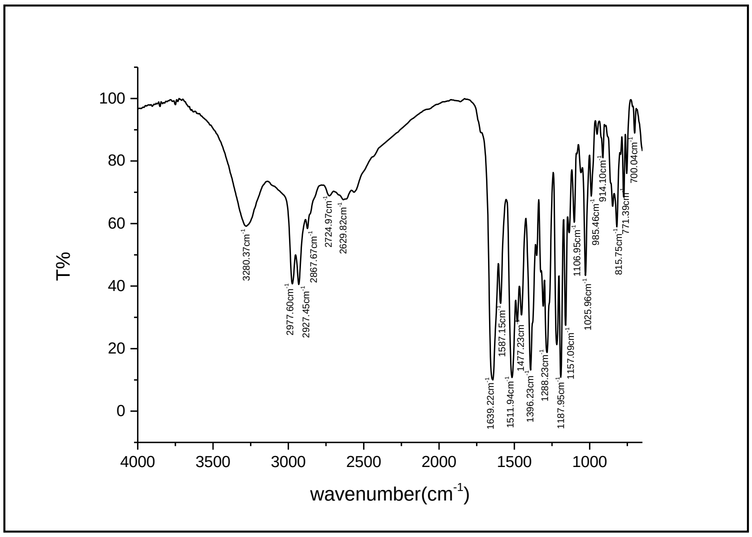

Figure S2. IR Spectrum of compound **1**.

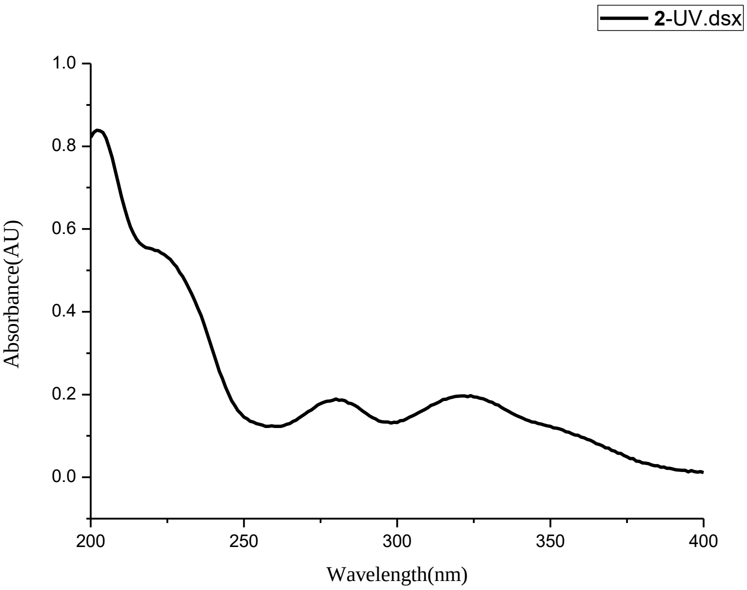

Figure S3. UV Spectrum of compound **1**.

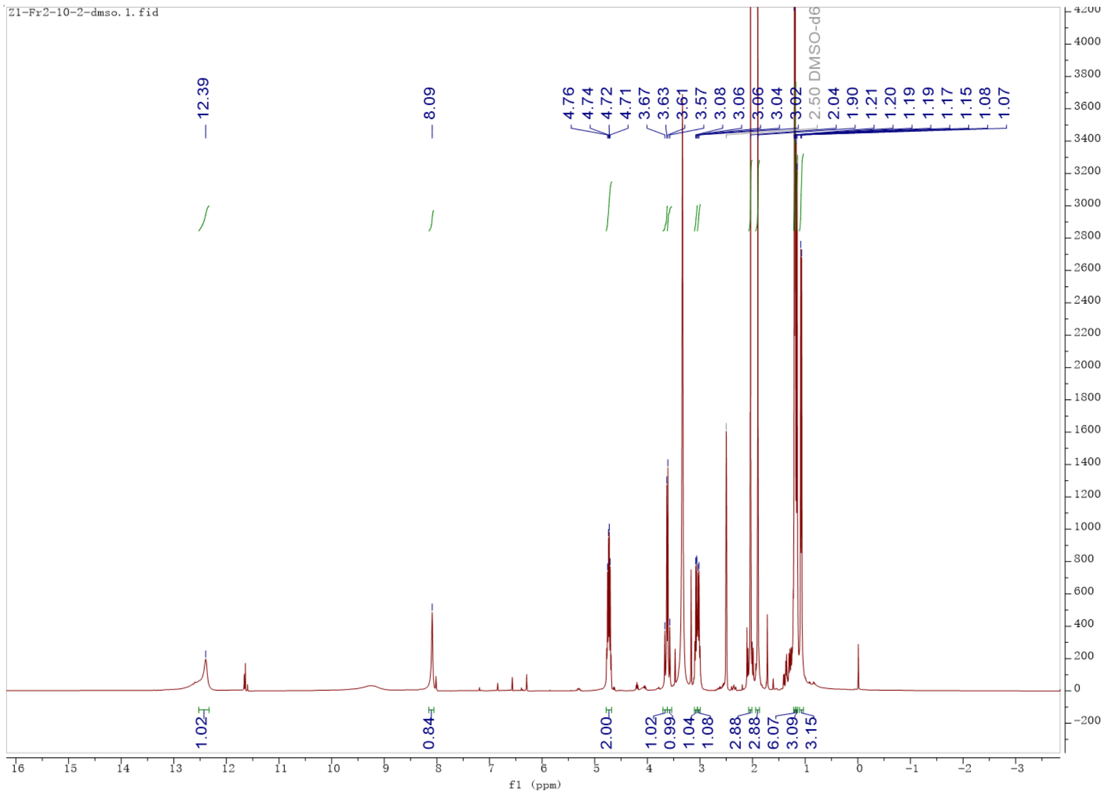

Figure S4. ^{1}H NMR (400 MHz) Spectrum of compound **1** in DMSO-d_6.

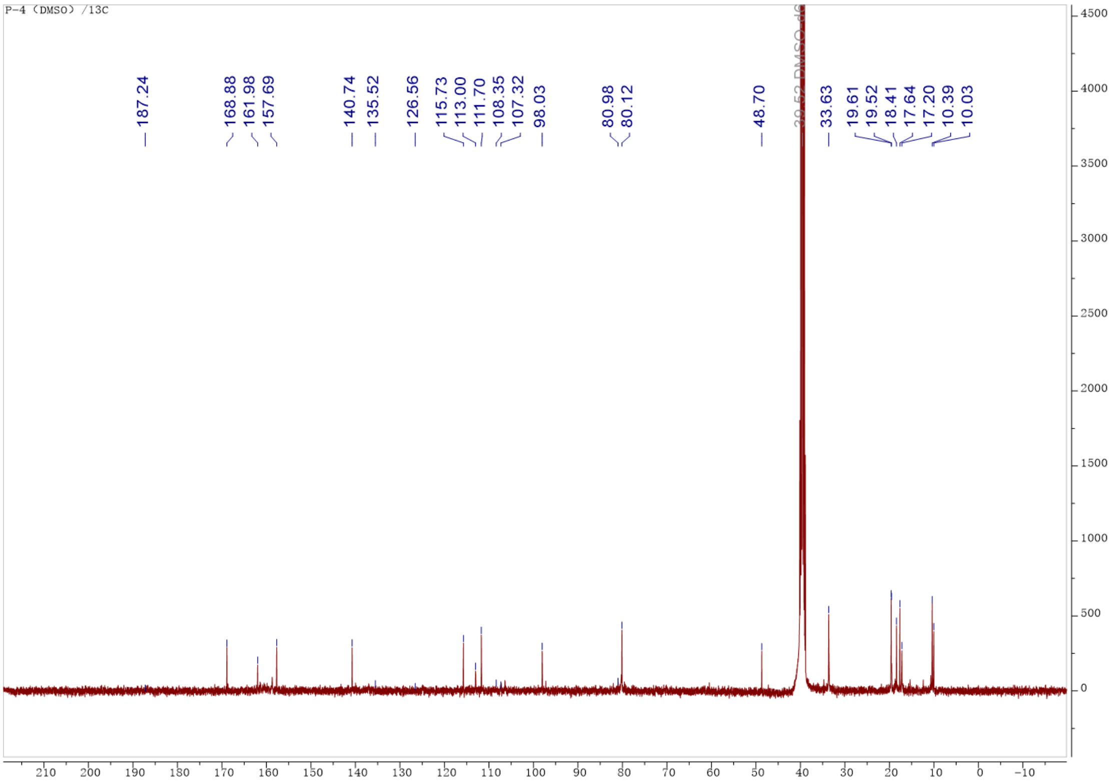

Figure S5. ^{13}C NMR (100 MHz) Spectrum of compound **1** in DMSO-d_6.

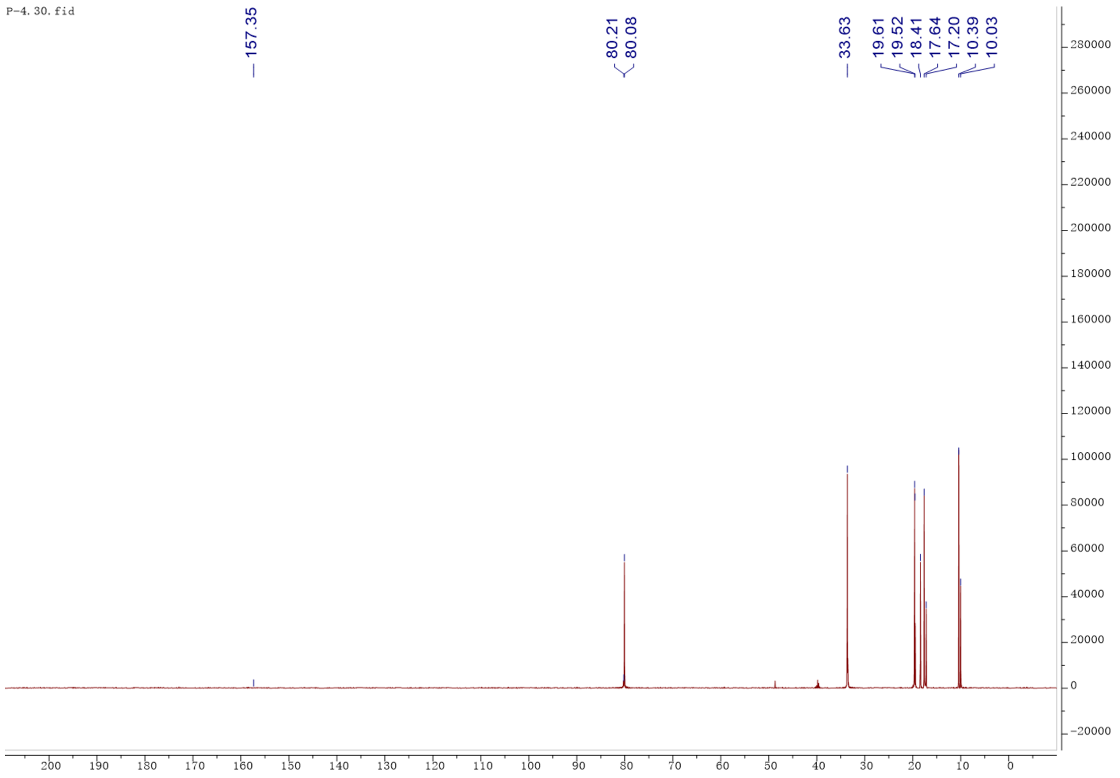

Figure S6. DEPT 135 (100 MHz) Spectrum of compound **1** in DMSO-d_6.

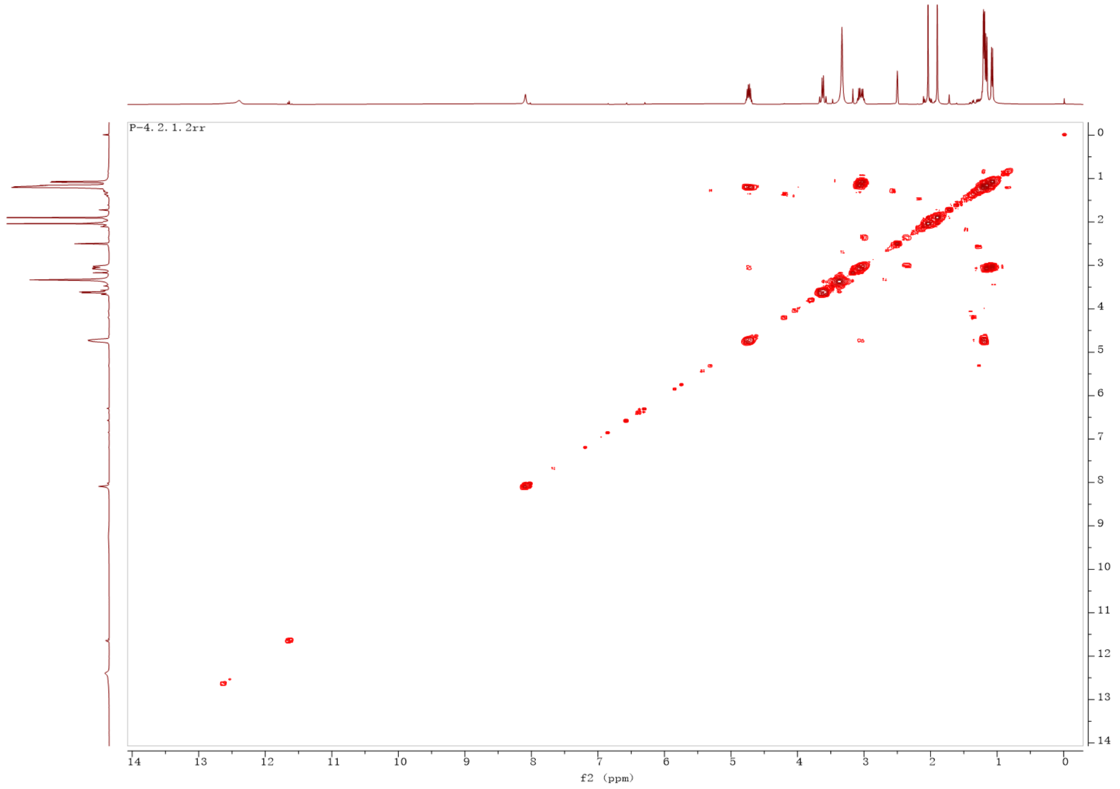

Figure S7. COSY Spectrum of compound **1** in DMSO-d_6.

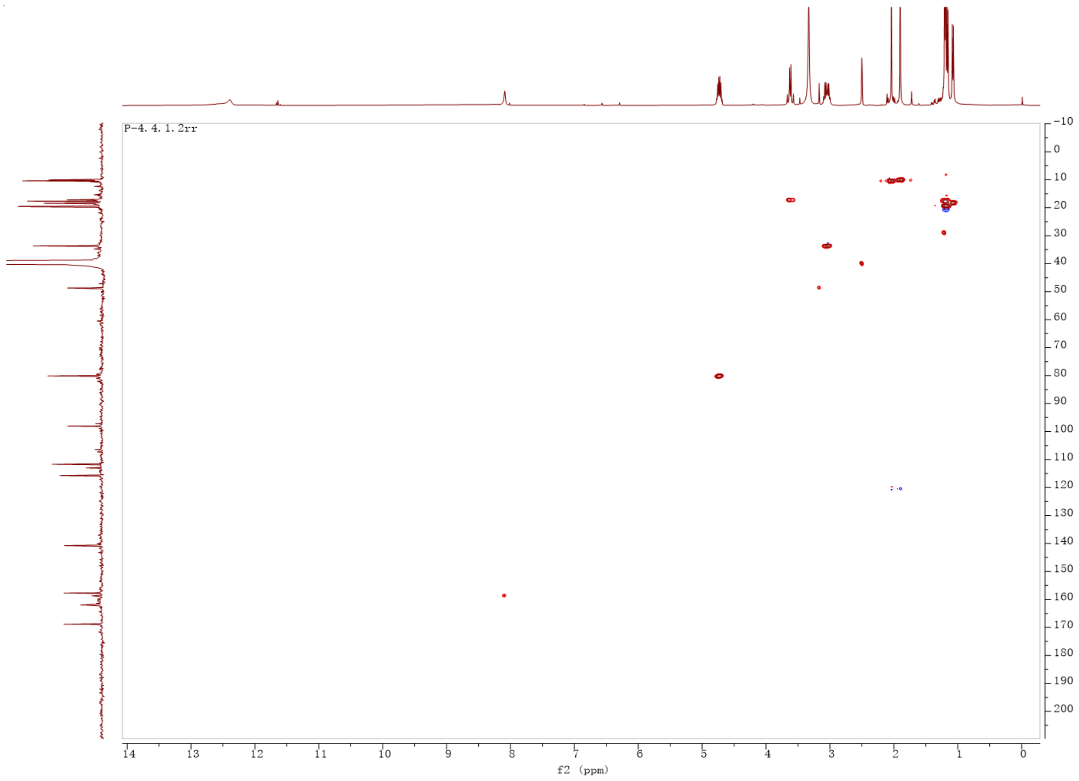

Figure S8. HSQC Spectrum of compound **1** in DMSO-d_6.

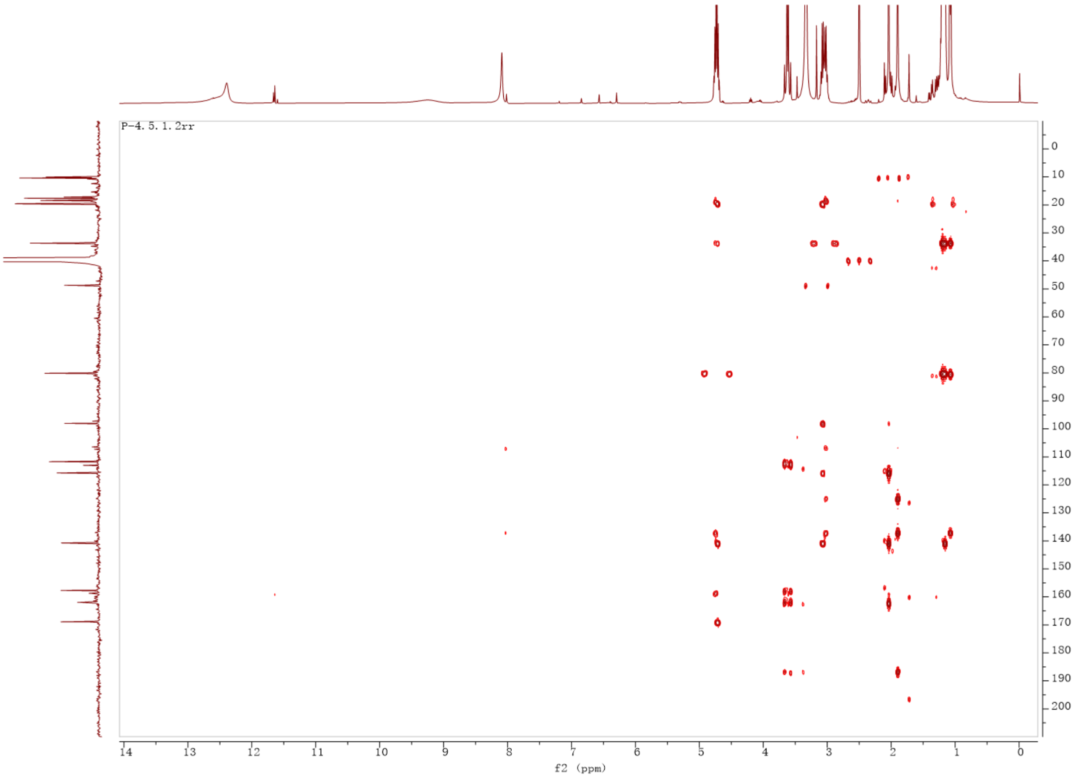

Figure S9. HMBC Spectrum of compound **1** in DMSO-d_6.

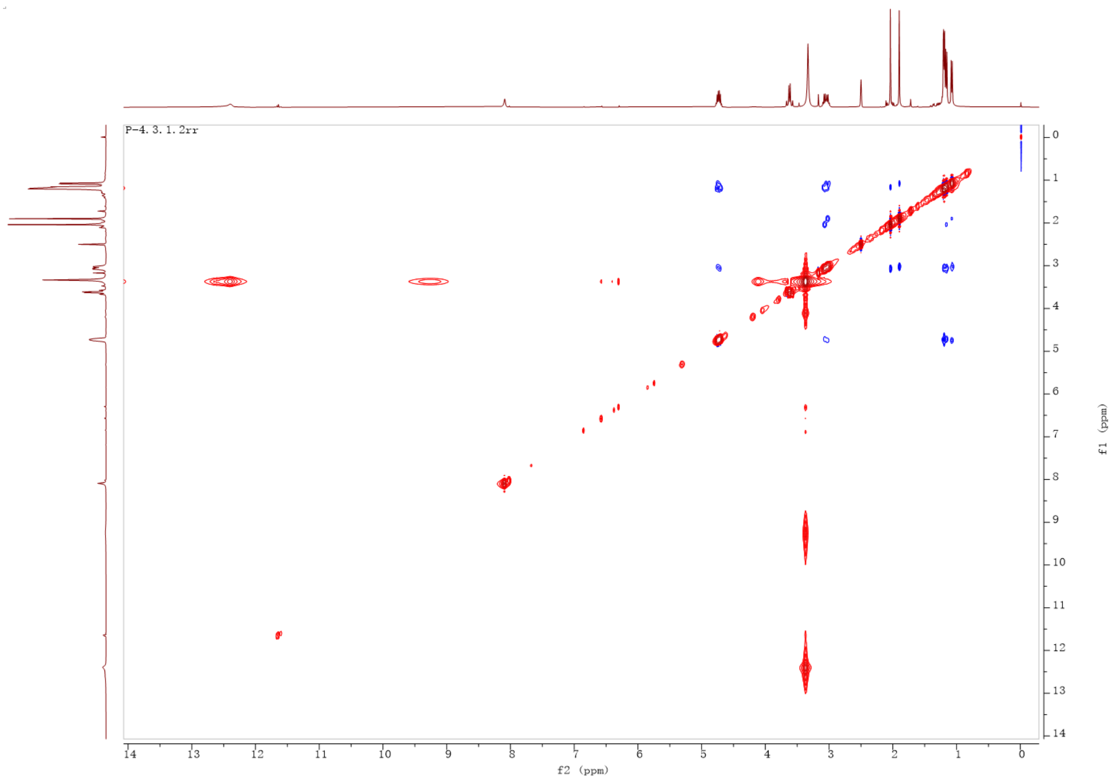

Figure S10. NOESY Spectrum of compound **1** in DMSO-d_6.

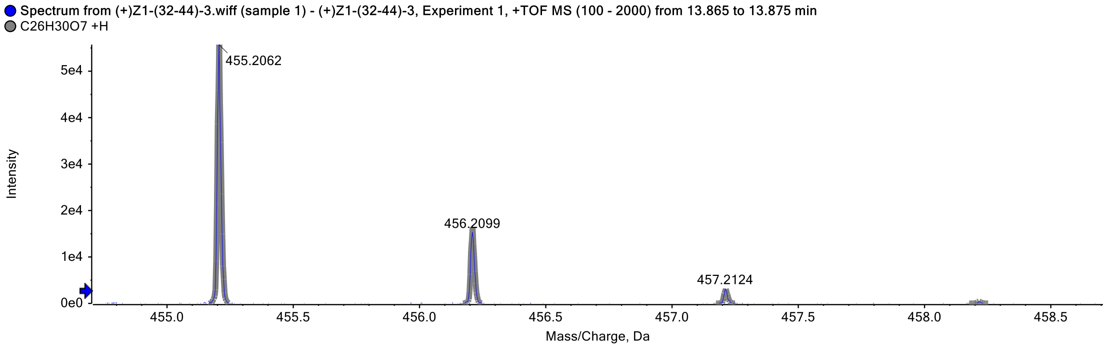

Figure S11. HRESIMS Spectrum of compound **2**.

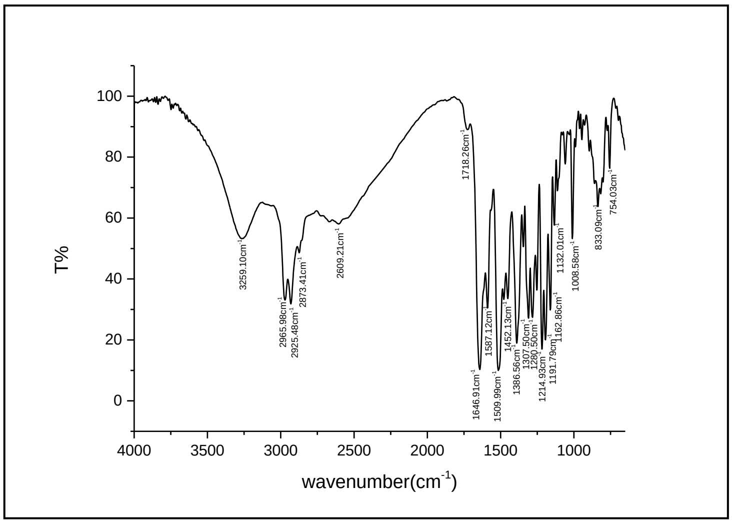

Figure S12. IR Spectrum of compound **2**.

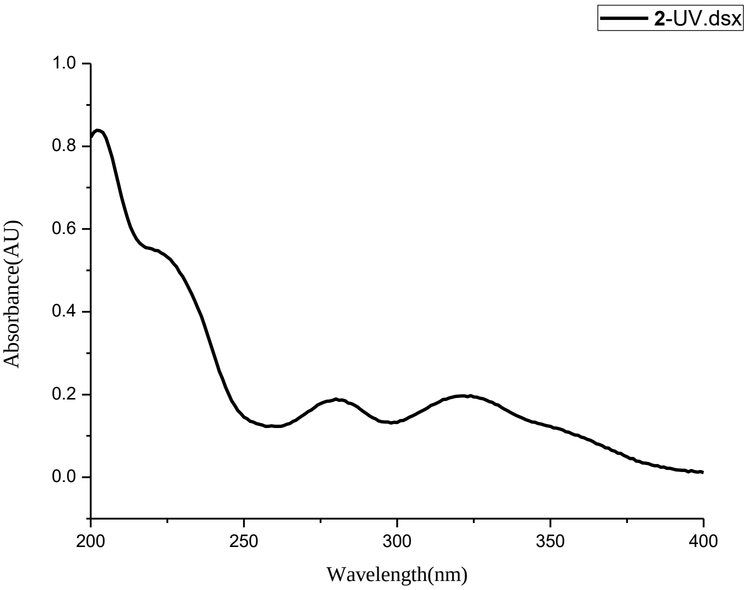

Figure S13. UV Spectrum of compound **2**.

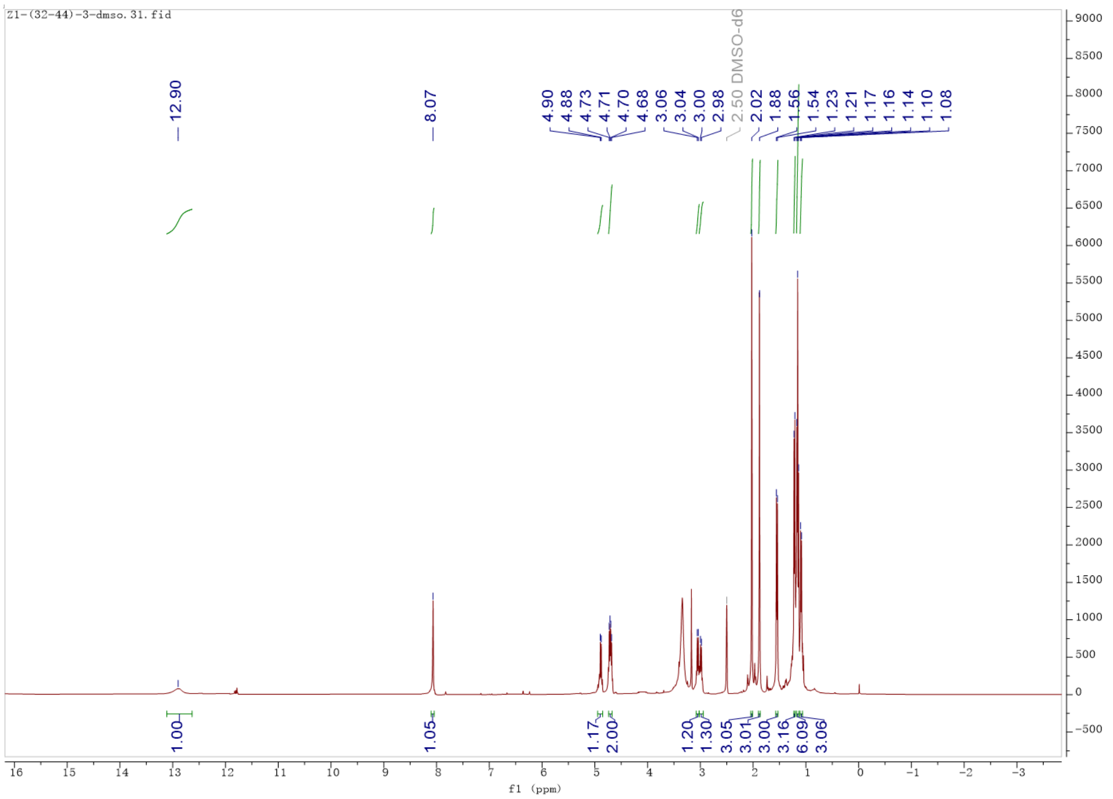

Figure S14. ^{1}H NMR (400 MHz) Spectrum of compound **2** in DMSO-d_6.

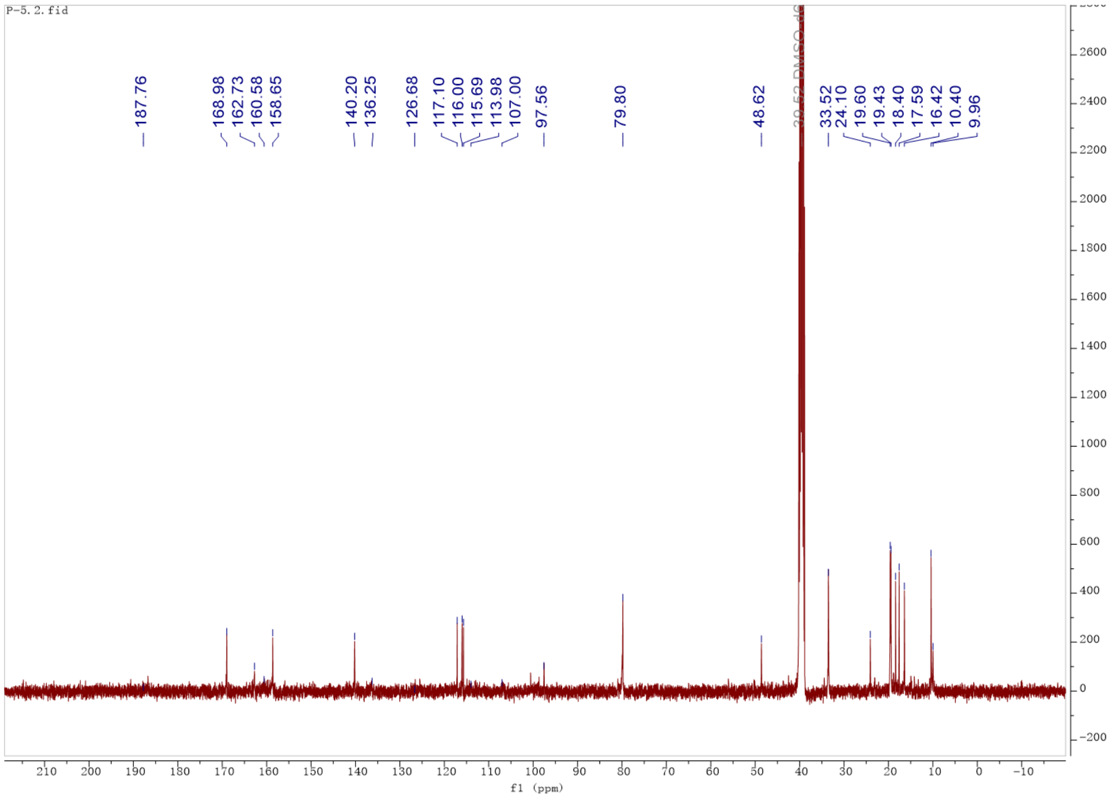

Figure S15. ^{13}C NMR (100 MHz) Spectrum of compound **2** in DMSO-d_6.

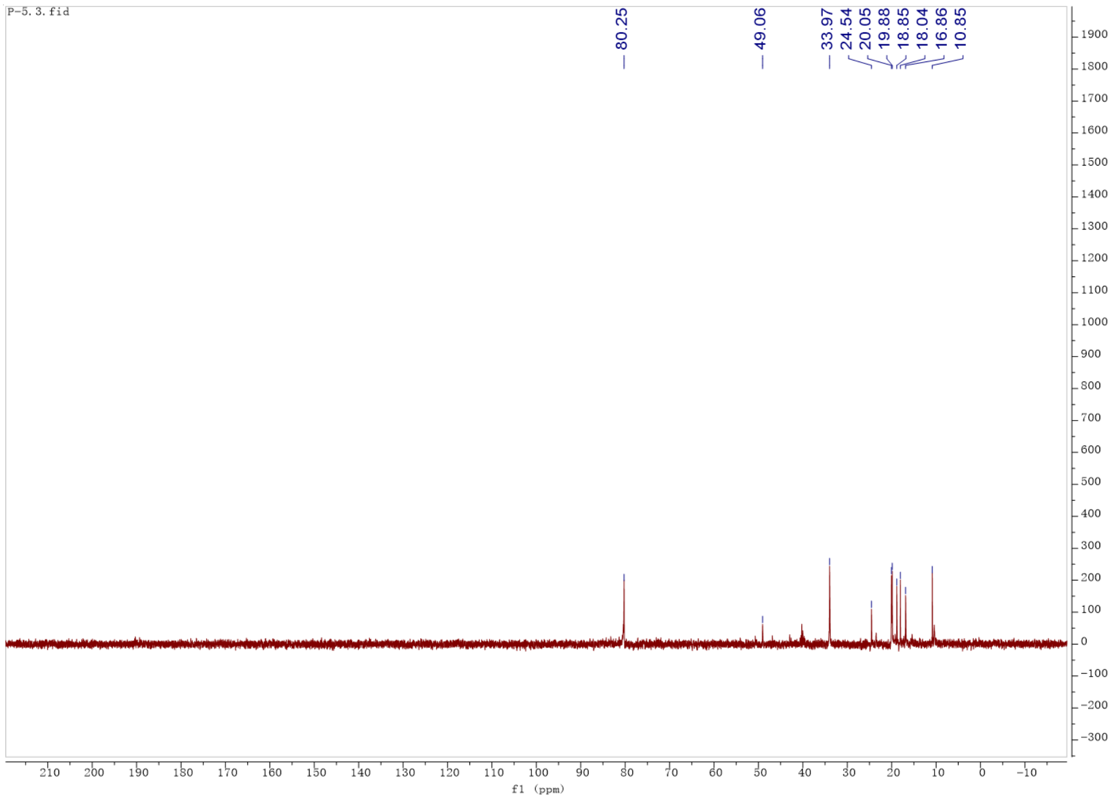

Figure S16. DEPT 135 (100 MHz) Spectrum of compound **2** in DMSO-d_6.

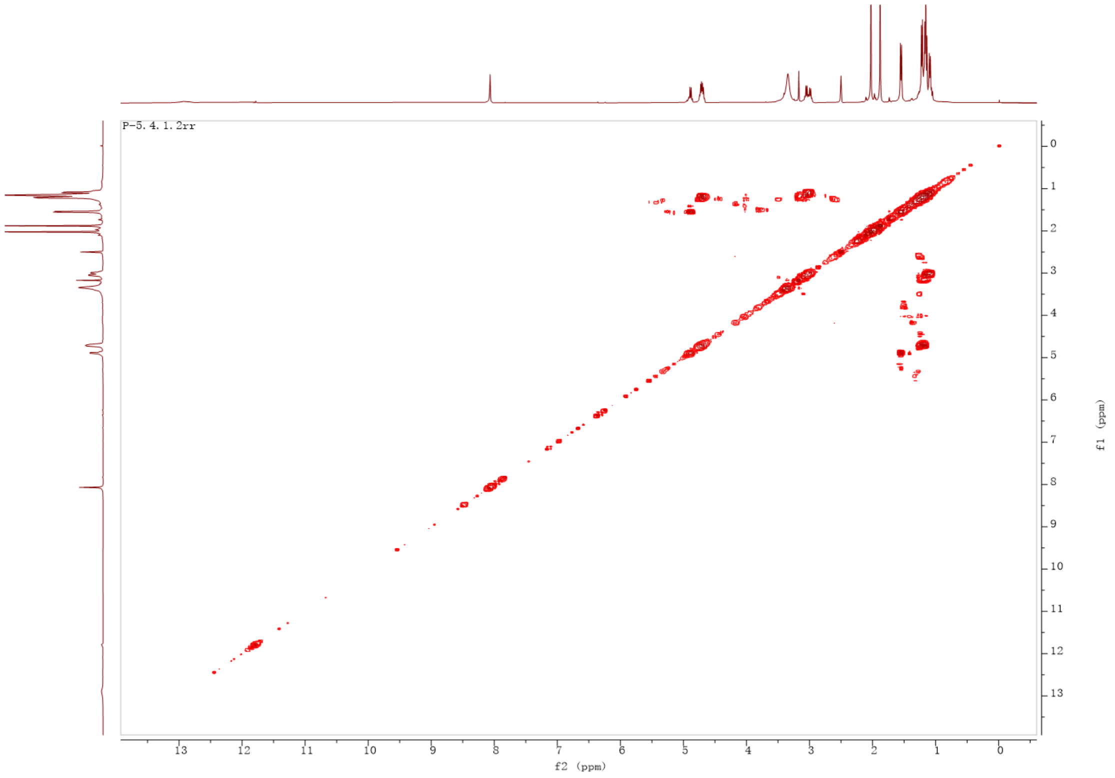

Figure S17. COSY Spectrum of compound **2** in DMSO-d_6.

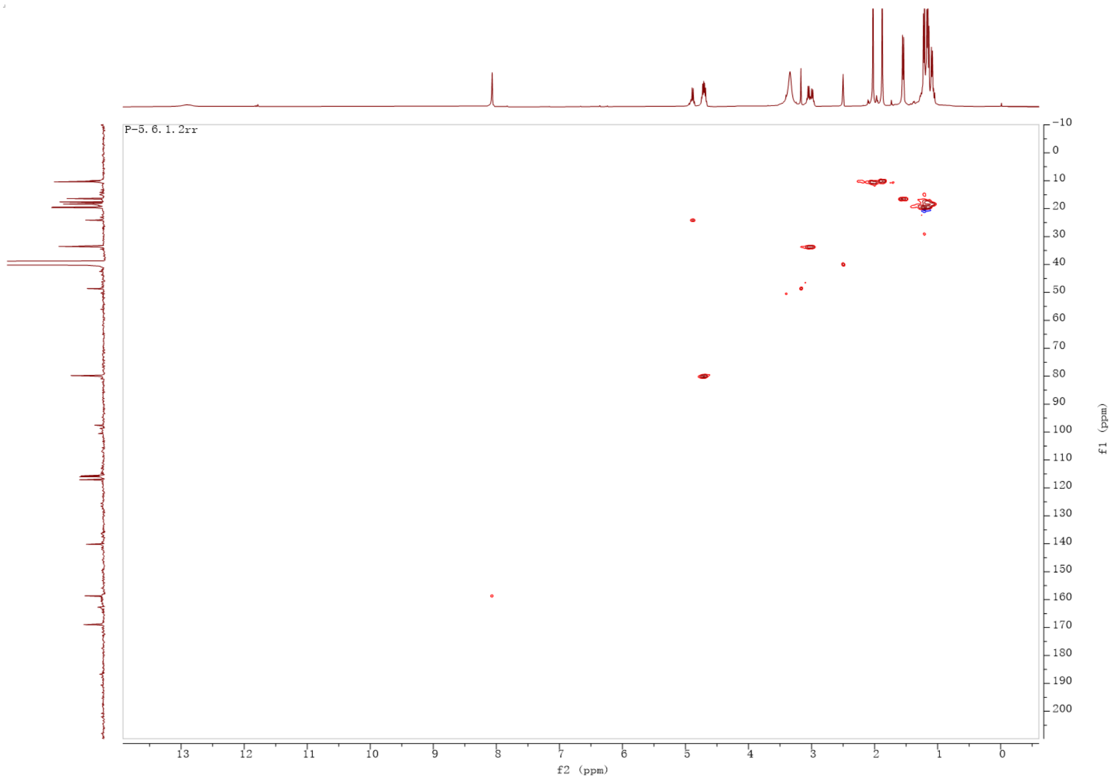

Figure S18. HSQC Spectrum of compound **2** in DMSO-d_6.

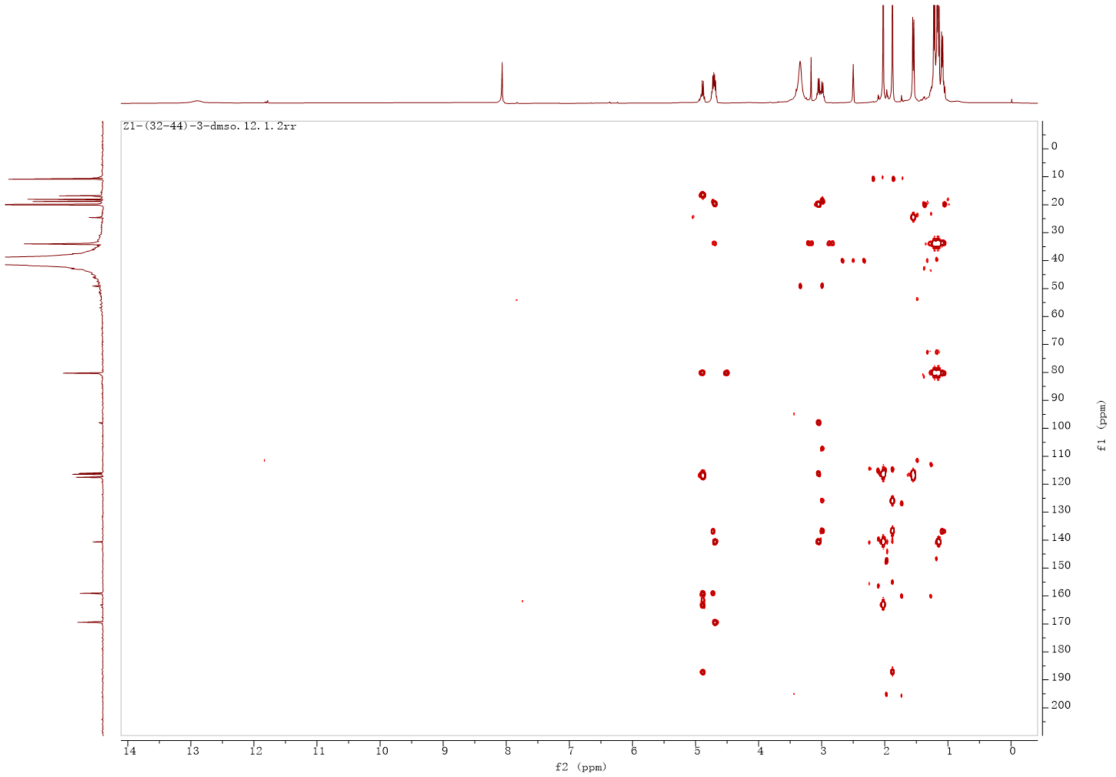

Figure S19. HMBC Spectrum of compound **2** in DMSO-d_6.

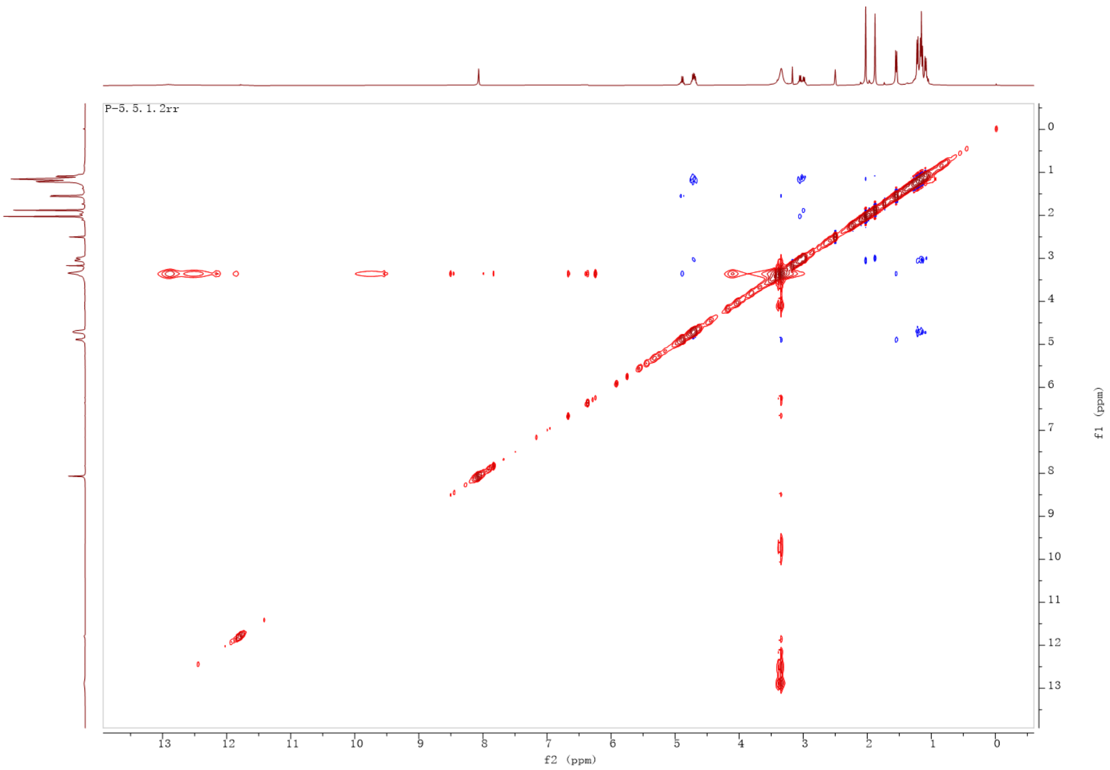

Figure S20. NOESY Spectrum of compound **2** in DMSO-d_6.

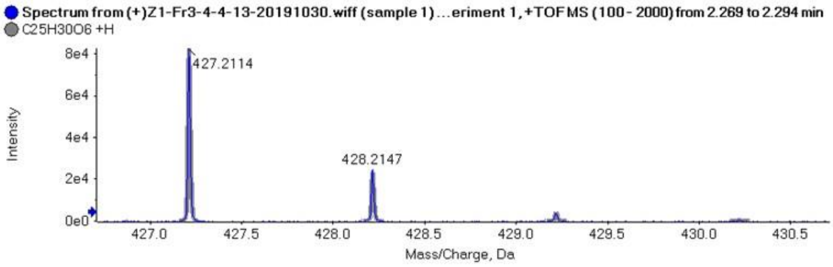

Figure S21. HRESIMS Spectrum of compound **3**.

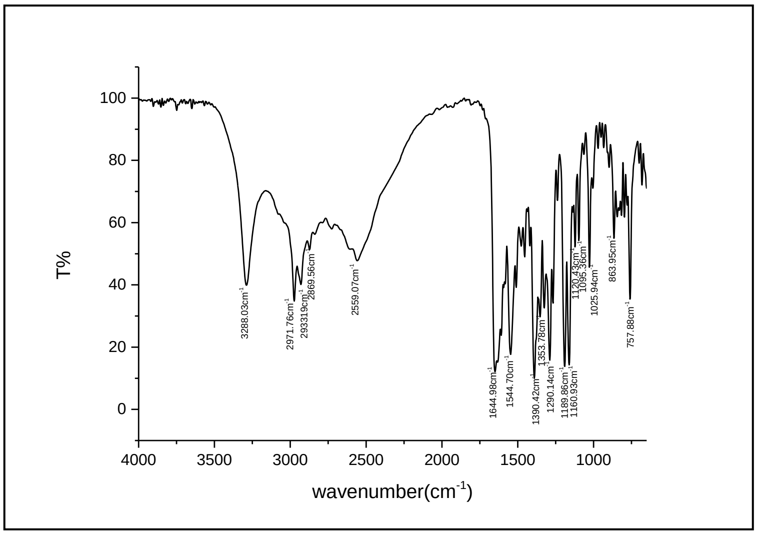

Figure S22. IR Spectrum of compound **3**.

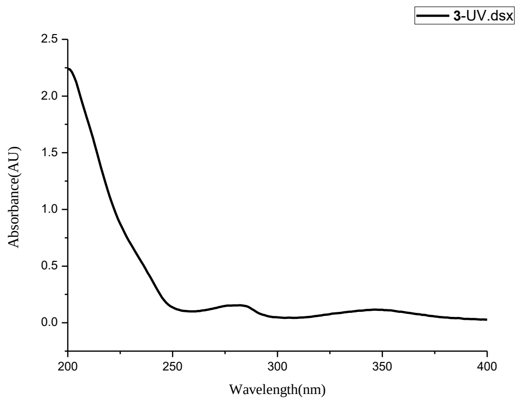

Figure S23. UV Spectrum of compound **3**.

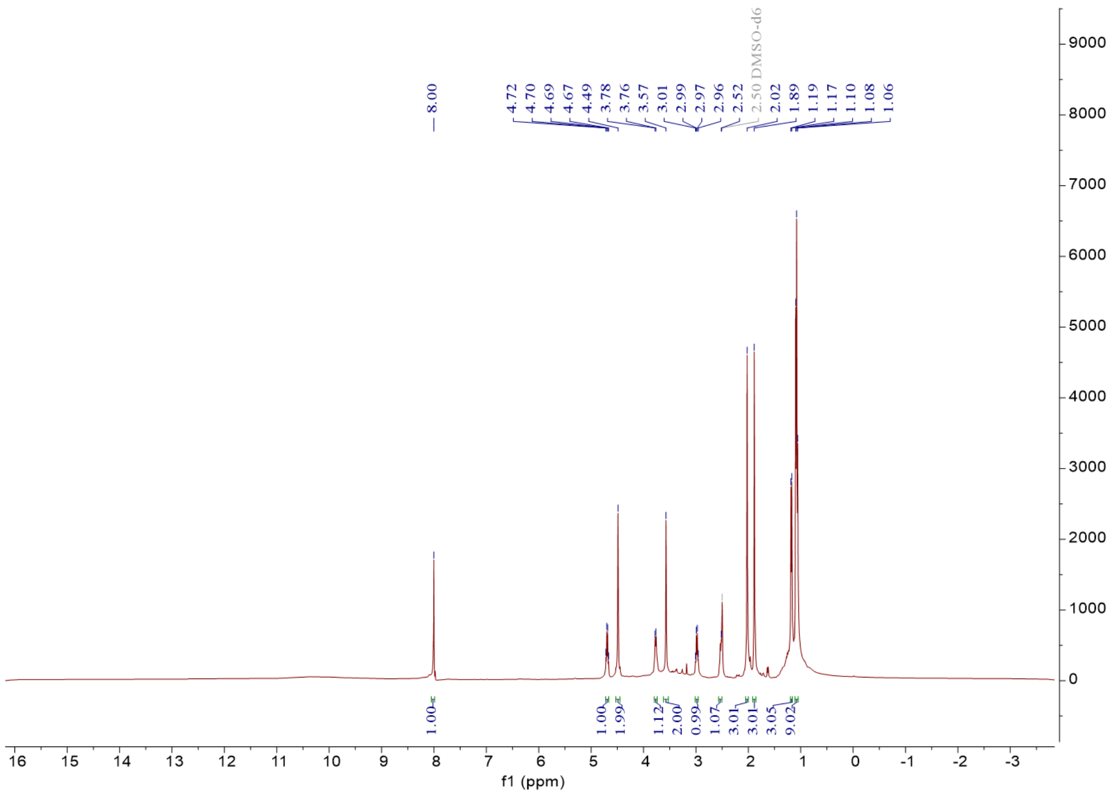

Figure S24. ^{1}H NMR (400 MHz) Spectrum of compound **3** in DMSO-d_6.

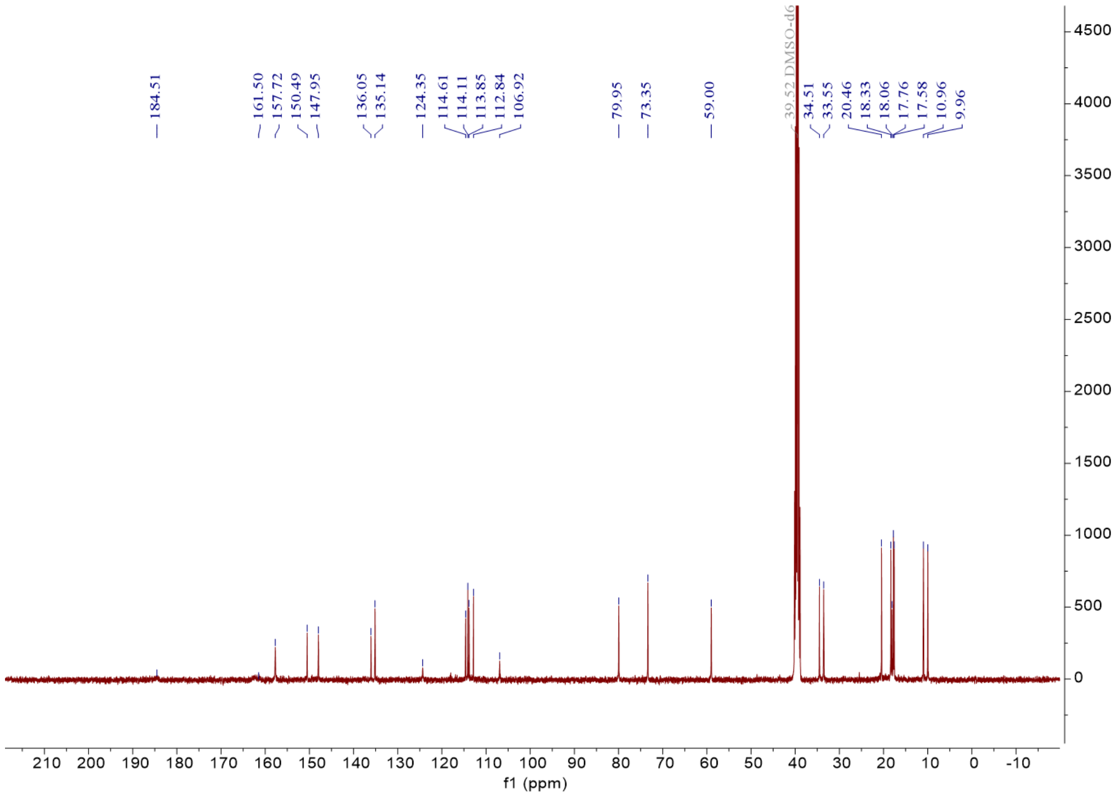

Figure S25. ^{13}C NMR (100 MHz) Spectrum of compound **3** in DMSO-*d*$_6$.

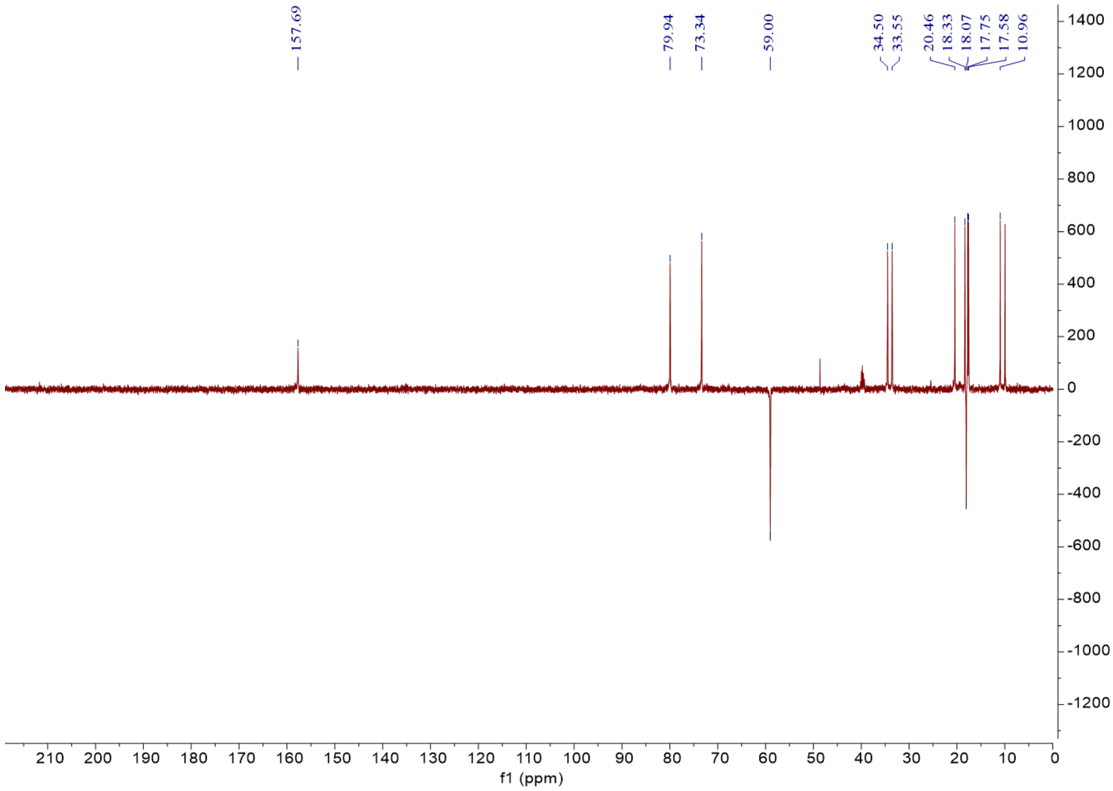

Figure S26. DEPT 135 (100 MHz) Spectrum of compound **3** in DMSO-*d*$_6$.

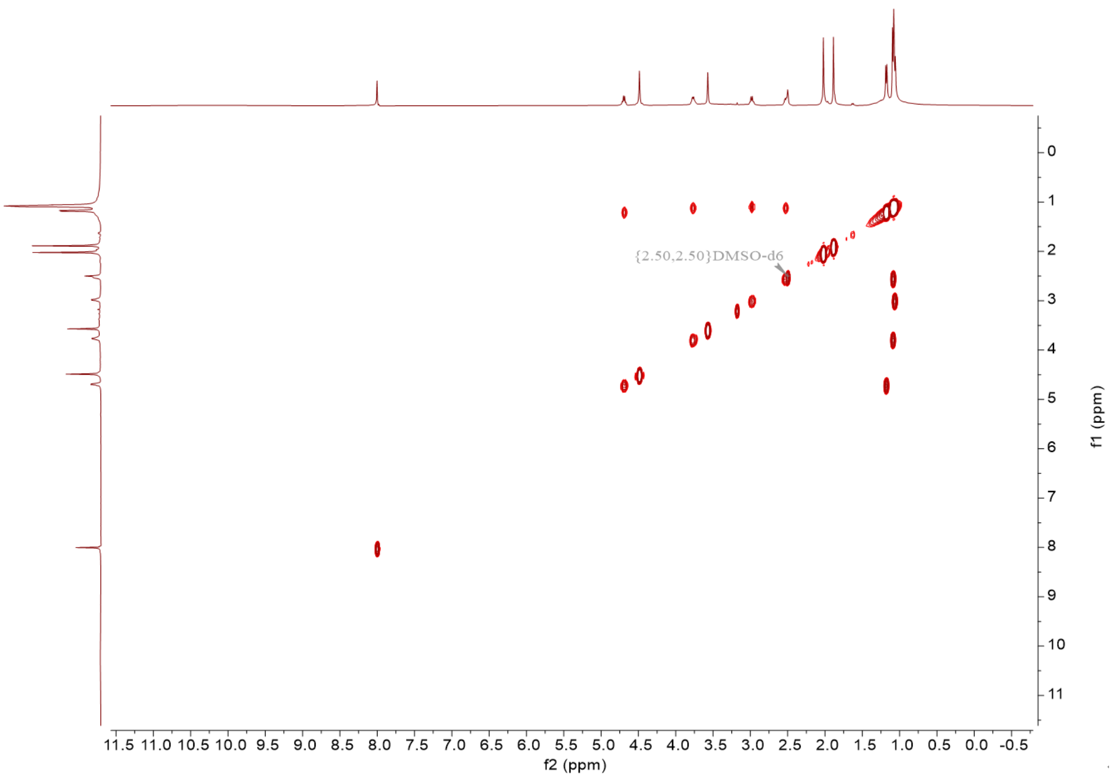

Figure S27. COSY Spectrum of compound **3** in DMSO-d_6.

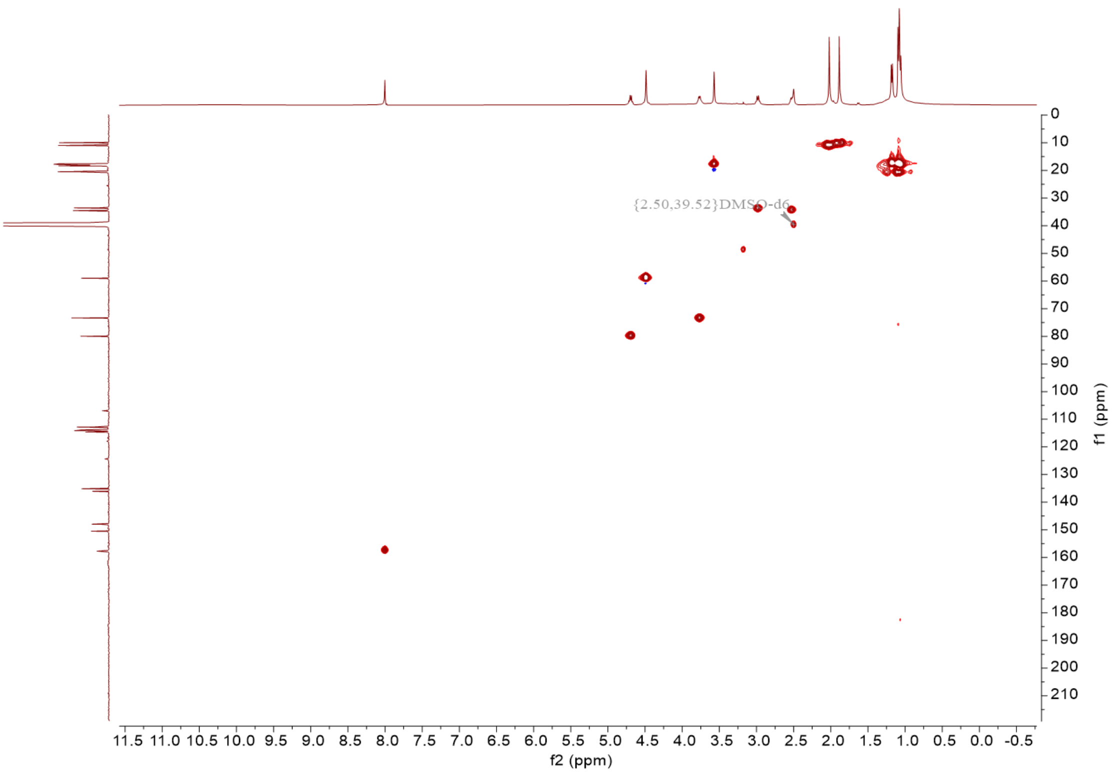

Figure S28. HSQC Spectrum of compound **3** in DMSO-d_6.

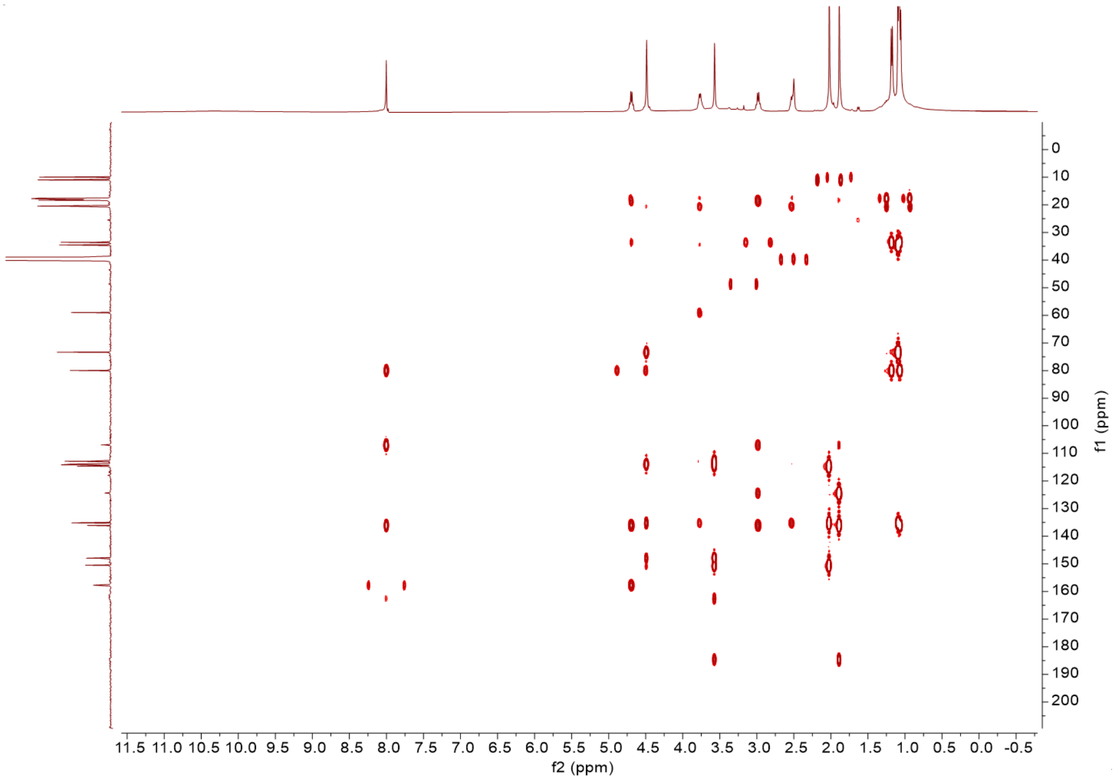

Figure S29. HMBC Spectrum of compound **3** in DMSO-d_6.

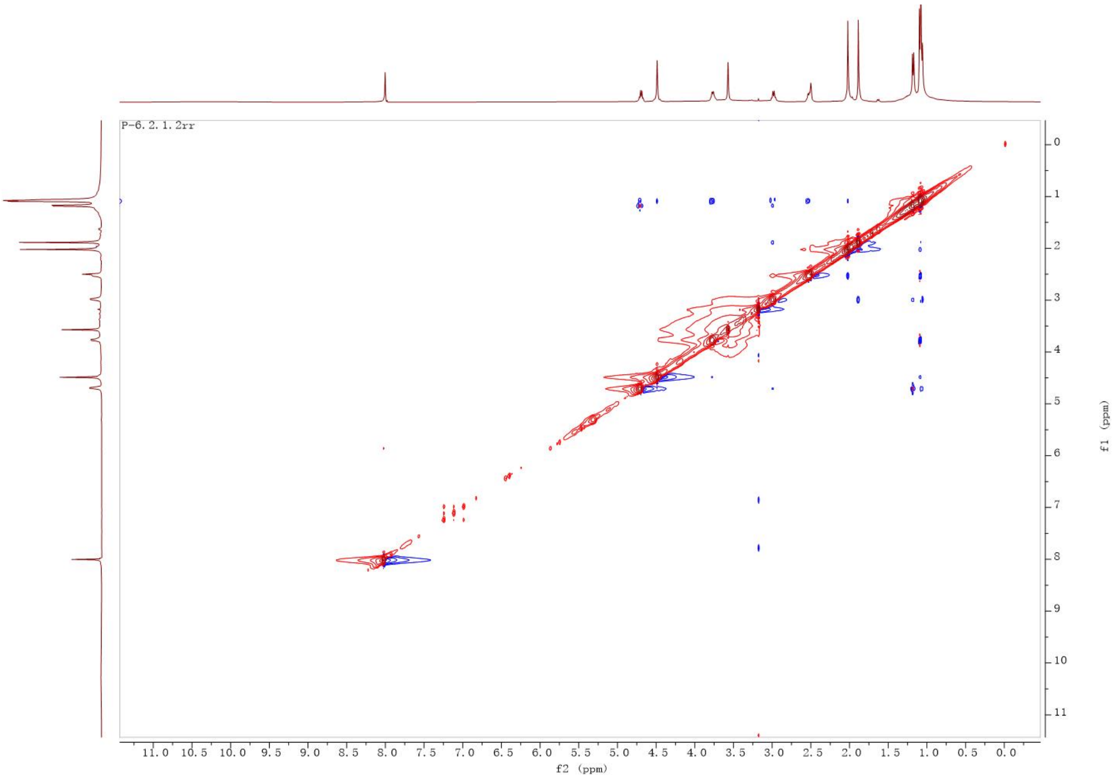

Figure S30. NOESY Spectrum of compound **3** in DMSO-d_6.

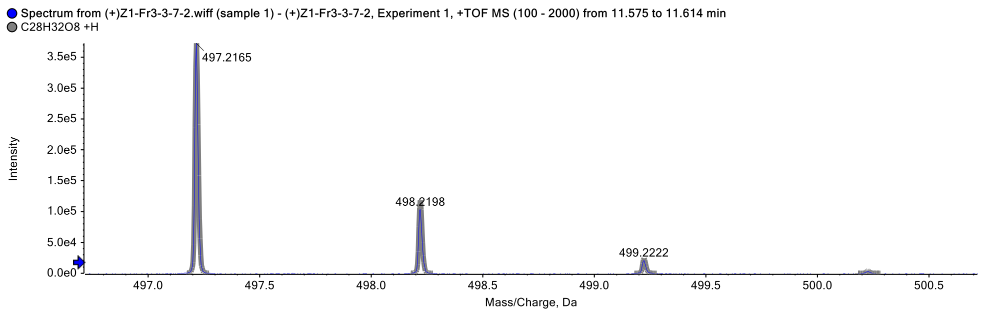

Figure S31. HRESIMS Spectrum of compound **4**.

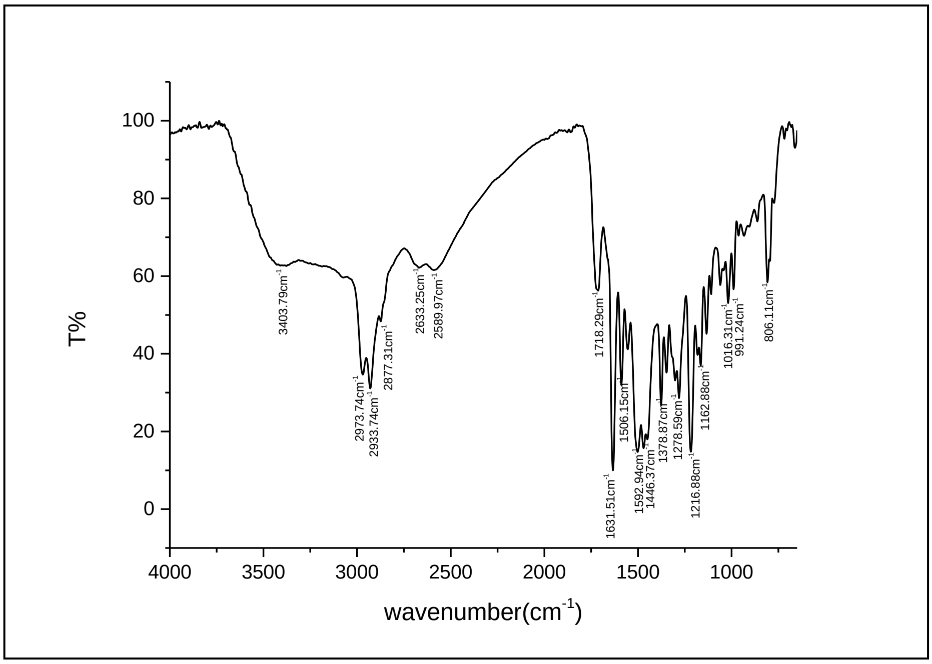

Figure S32. IR Spectrum of compound **4**.

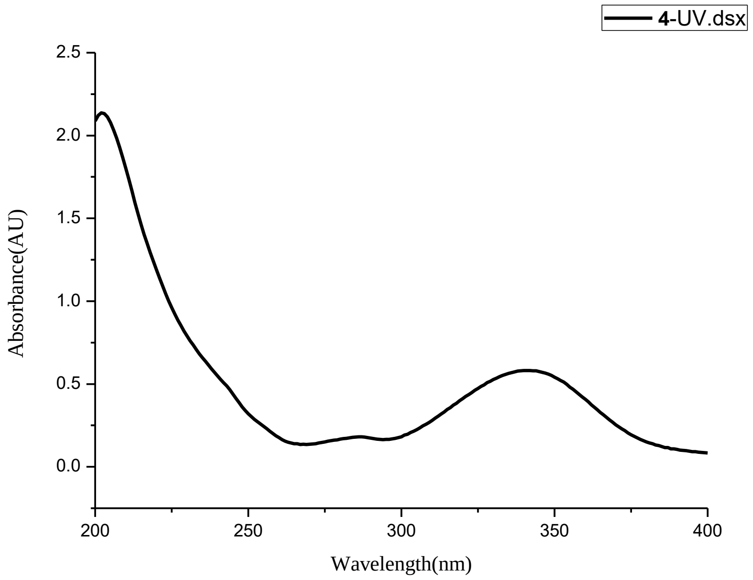

Figure S33. UV Spectrum of compound **4**.

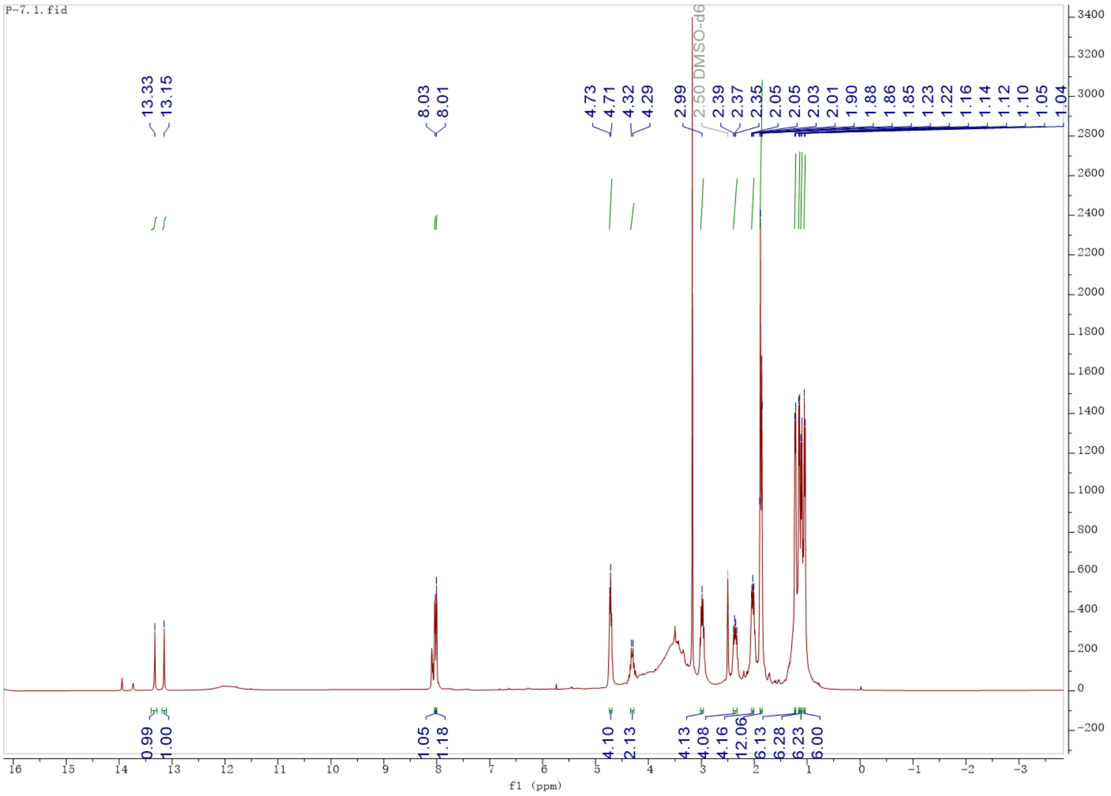

Figure S34. ^{1}H NMR (400 MHz) Spectrum of compound **4** in DMSO-d_6.

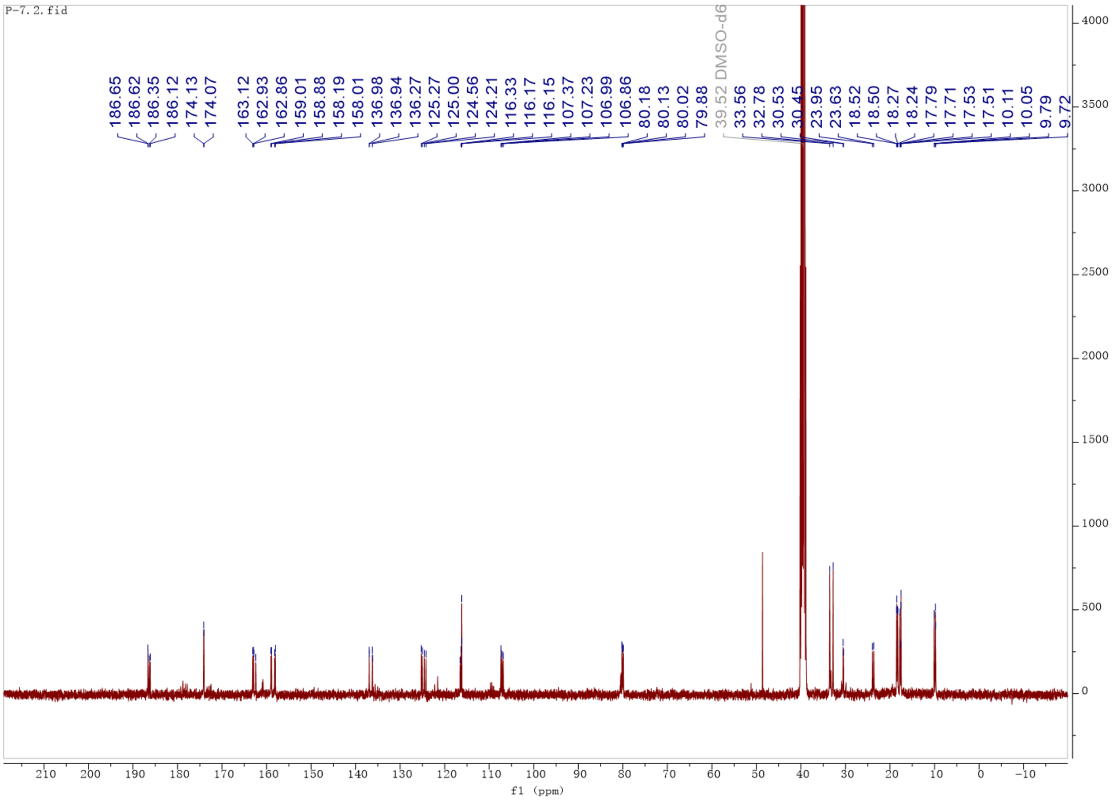

Figure S35. ^{13}C NMR (100 MHz) Spectrum of compound **4** in DMSO-d_6.

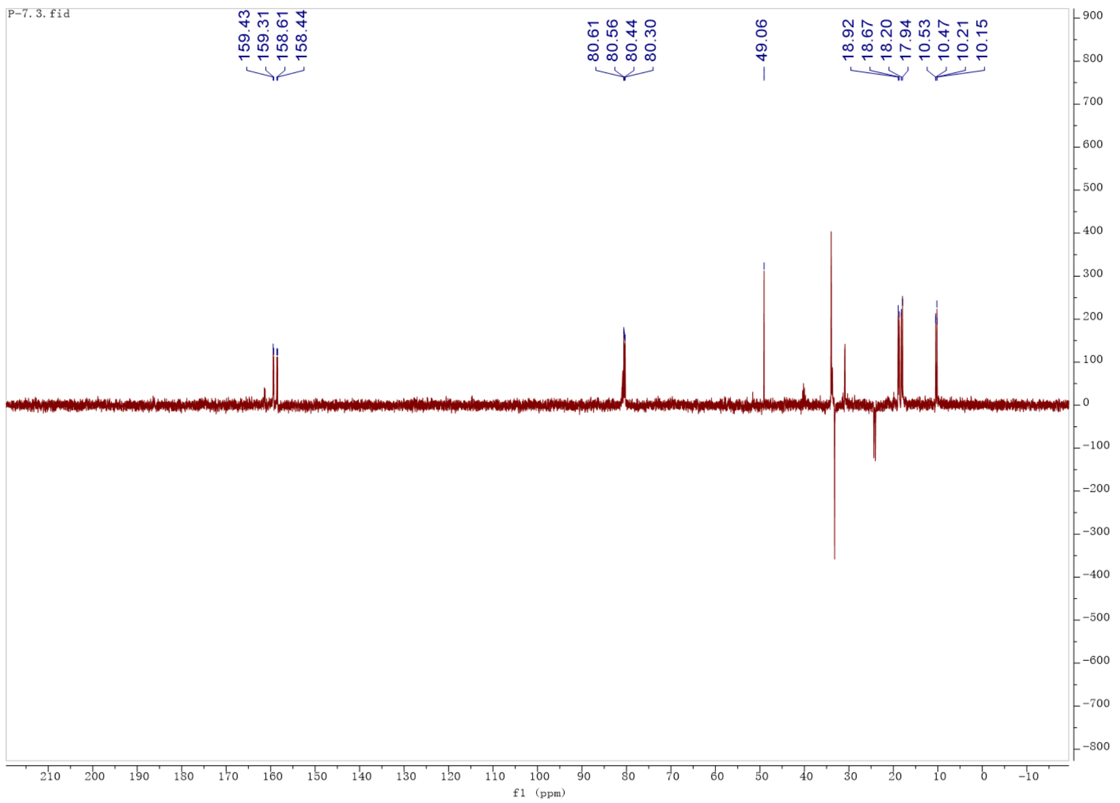

Figure S36. DEPT 135 (100 MHz) Spectrum of compound **4** in DMSO-d_6.

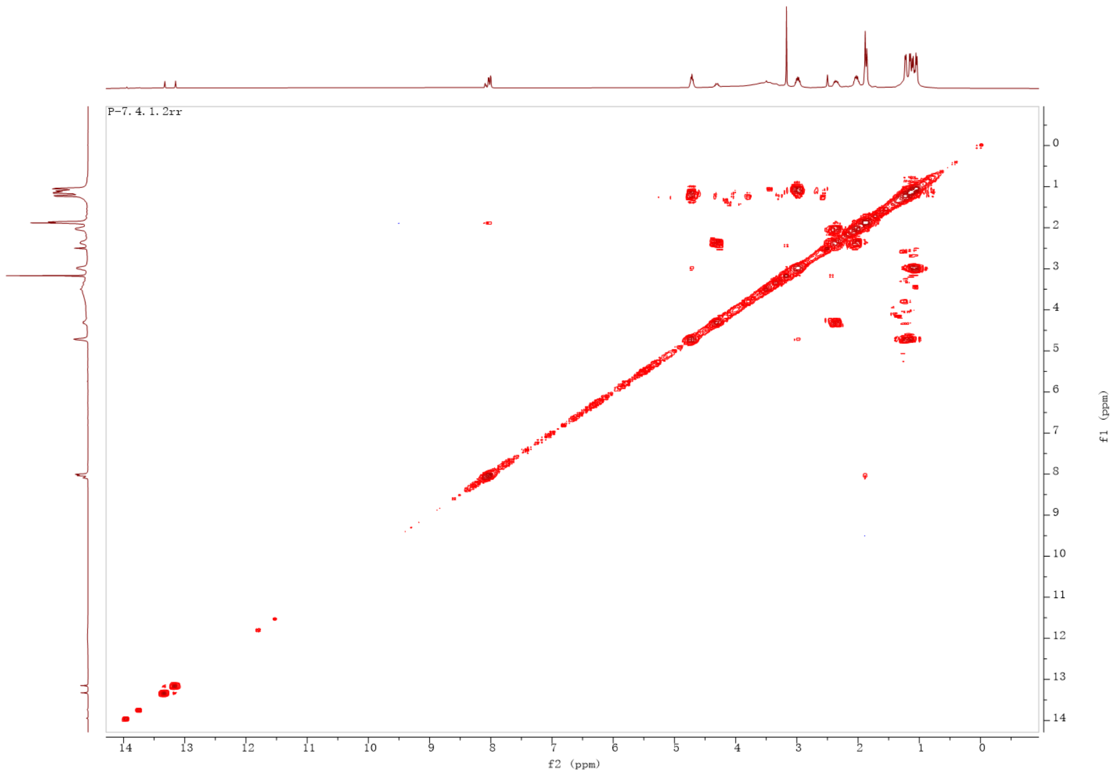

Figure S37. COSY Spectrum of compound **4** in DMSO-d_6.

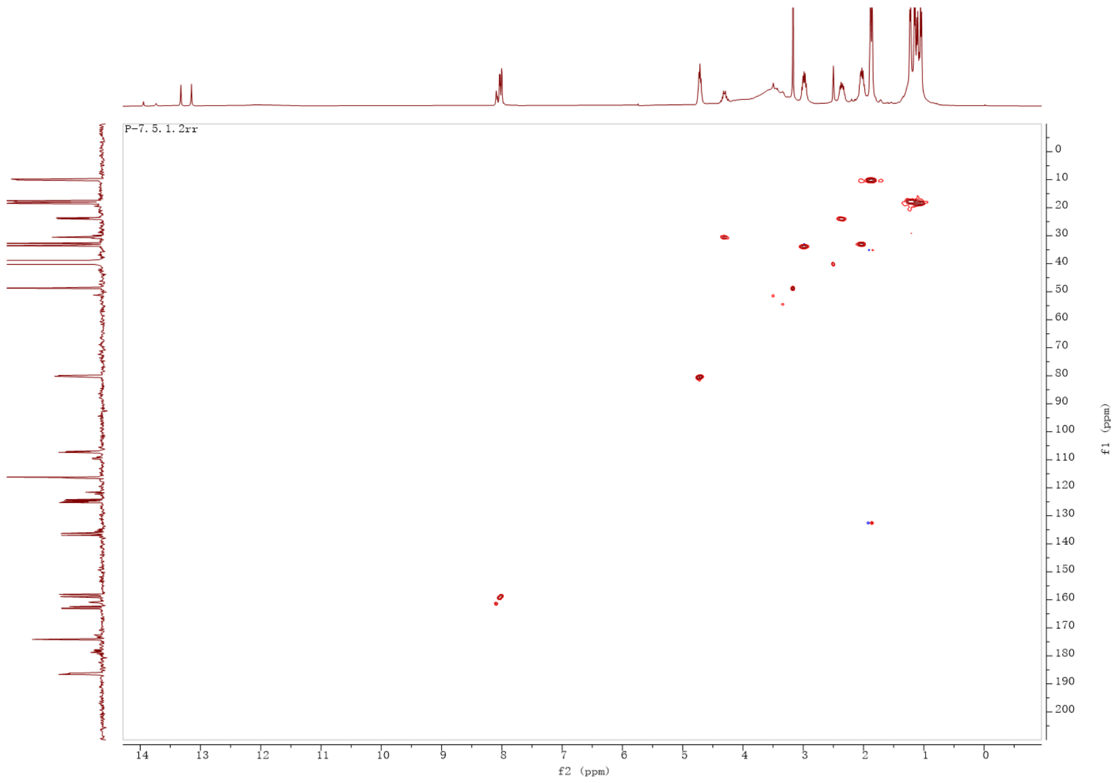

Figure S38. HSQC Spectrum of compound **4** in DMSO-d_6.

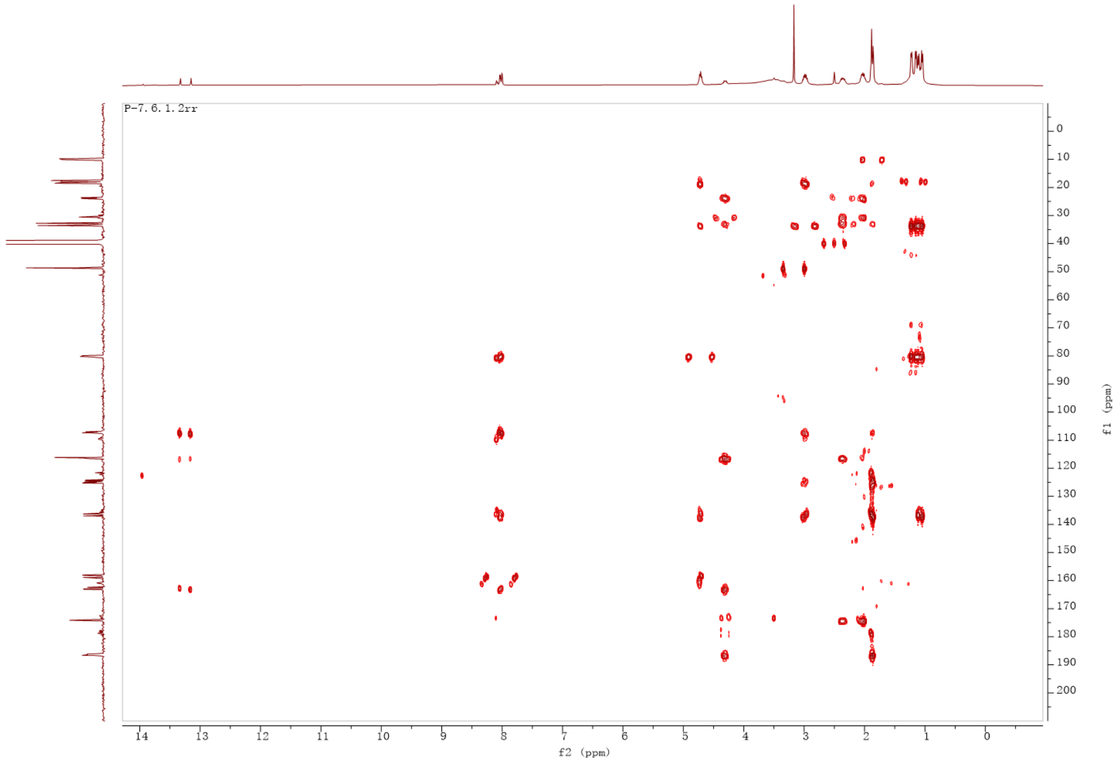

Figure S39. HMBC Spectrum of compound **4** in DMSO-d_6.

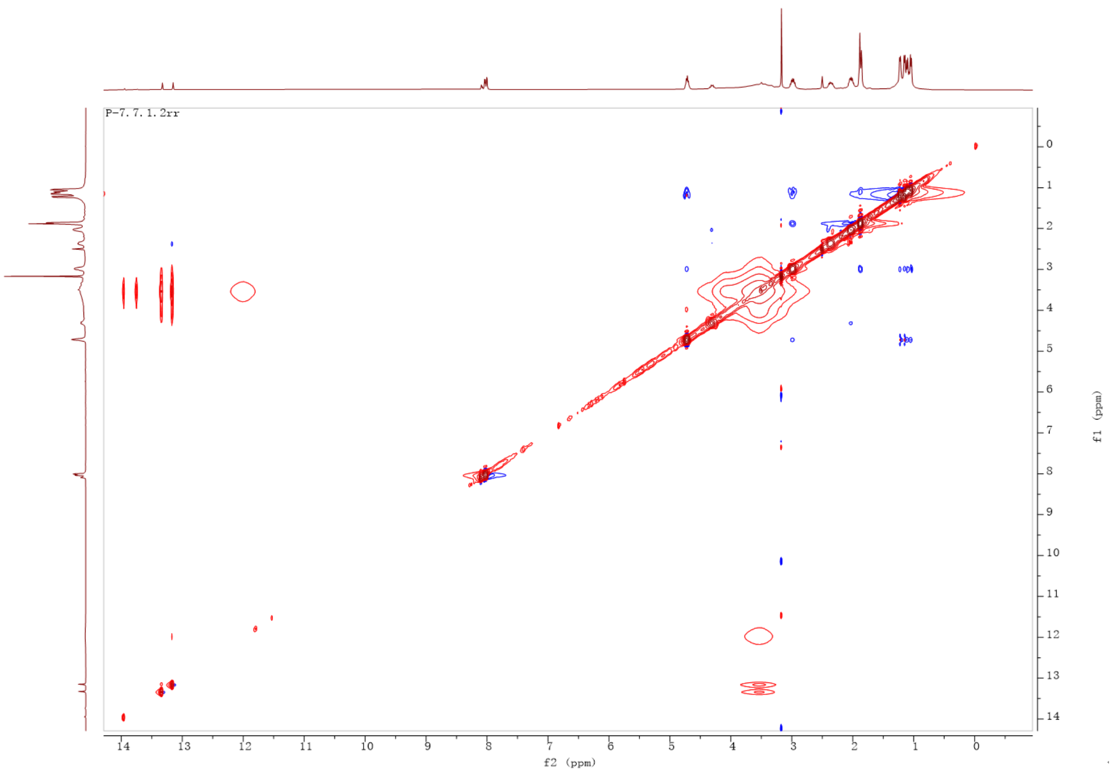

Figure S40. NOESY Spectrum of compound **4** in DMSO-d_6.

Table S1. Cartesian coordinate of dominant conformer of **1** (B3LYP/6-31+g(d))

Standard orientation					
Center Number	Atomic Number	Atomic Type	Coordinates (Angstroms)		
			X	Y	Z
1	6	0	-3.32084	1.550404	0.073524
2	6	0	-3.82526	0.318446	0.326405
3	6	0	-3.14477	-0.89271	-0.12939
4	6	0	-1.87274	-0.72251	-0.81908
5	6	0	-1.34851	0.503973	-1.12207
6	6	0	-2.02245	1.705913	-0.64282
7	6	0	-5.07361	0.09283	1.156057
8	6	0	-5.81996	-1.18505	0.727661
9	8	0	-4.90656	-2.34526	0.589572
10	6	0	-3.68503	-2.12901	0.081691
11	6	0	1.161949	0.6192	-1.17606
12	6	0	1.908477	-0.56849	-1.10643
13	6	0	3.119653	-0.65653	-0.38787
14	6	0	3.64518	0.513518	0.22423
15	6	0	2.949103	1.710455	0.1744
16	6	0	1.689623	1.748583	-0.51616
17	6	0	3.818602	-1.92829	-0.30956

18	8	0	4.912396	-2.06458	0.50468
19	6	0	5.200886	-1.01533	1.487023
20	6	0	4.995181	0.395612	0.89855
21	6	0	4.34133	-1.28144	2.711833
22	6	0	-4.69124	0.023411	2.638102
23	6	0	-6.54378	-1.04209	-0.59831
24	6	0	6.097093	0.729833	-0.11112
25	8	0	-1.53712	2.833286	-0.80318
26	8	0	3.502341	-2.93342	-0.94393
27	8	0	1.40706	-1.64119	-1.78195
28	8	0	1.079103	2.939094	-0.50933
29	6	0	3.456346	2.970039	0.795659
30	6	0	-3.97766	2.817567	0.506948
31	8	0	-1.31267	-1.91049	-1.15864
32	6	0	-0.11583	0.680945	-1.961
33	1	0	-5.77682	0.955575	1.007612
34	1	0	-6.48629	-1.57277	1.532611
35	1	0	-3.1967	-3.08828	-0.12271
36	1	0	6.278557	-1.2293	1.680375
37	1	0	5.050625	1.129469	1.747346
38	1	0	4.689522	-0.72057	3.585727
39	1	0	3.287797	-1.01821	2.545208
40	1	0	4.361	-2.34986	2.97334
41	1	0	-5.57369	-0.02304	3.284948
42	1	0	-4.10948	0.905506	2.938919
43	1	0	-4.06967	-0.85566	2.853786
44	1	0	-5.89366	-0.6291	-1.38323
45	1	0	-6.90149	-2.01587	-0.96221
46	1	0	-7.41624	-0.38256	-0.5162
47	1	0	6.147707	-0.02244	-0.91102
48	1	0	5.916517	1.700573	-0.59067
49	1	0	7.082603	0.773691	0.36451
50	1	0	2.032887	-2.48138	-1.71914
51	1	0	0.09575	2.921729	-0.90492
52	1	0	3.944823	2.79058	1.761788
53	1	0	4.177737	3.472625	0.13608
54	1	0	2.636262	3.687384	0.972188
55	1	0	-4.0916	2.868791	1.597425
56	1	0	-3.37724	3.698578	0.210059
57	1	0	-4.96754	2.940907	0.049362

58	1	0	-0.37869	-1.80763	-1.57095
59	1	0	-0.09354	-0.08218	-2.773
60	1	0	-0.17599	1.658132	-2.49838

Table S2. Key transitions and their related rotatory and oscillator strengths of dominant conformer of **1** at the B3LYP/6-31+g(d) level.

HOMO is 117					
**No.**	**Energy (cm-1)**	**Wavelength (nm)**	**R(length)**	**Osc. Strength**	**Major contribs**
1	31188.653215	320.629426704	17.455	0.0242	H-3->LUMO (33%), H-2->LUMO (51%), HOMO->LUMO (10%)
2	32433.1666989	308.32635286	-79.4576	0.5649	H-2->LUMO (14%), HOMO->LUMO (81%)
3	35227.8777954	283.866092021	6.9974	0.0058	H-4->LUMO (76%)
4	36016.688027	277.6490718	-22.1853	0.1544	H-1->L+1 (78%)
5	38578.3048935	259.213048049	85.0454	0.2363	H-3->L+1 (13%), H-2->L+1 (32%), HOMO->L+1 (25%)
6	39820.3987142	251.127570866	3.6198	0.0238	H-1->LUMO (71%)
7	40354.3377462	247.804834833	6.743	0.0023	H-4->LUMO (11%), H-3->LUMO (49%), H-2->LUMO (19%), H-1->LUMO (10%)
8	44484.7029771	224.796375625	-3.9379	0.0338	H-5->L+1 (14%), H-3->L+1 (25%), HOMO->L+1 (39%)
9	45446.1158564	220.040806823	-28.3807	0.0218	H-5->L+1 (67%)
10	45882.4618025	217.948200841	-136.7155	0.343	HOMO->L+2 (17%), HOMO->L+4 (23%)
11	47043.0936259	212.571054096	-25.58	0.0036	H-6->LUMO (58%)
12	47310.0631419	211.371520896	-11.9451	0.0381	H-1->L+2 (10%), HOMO->L+2 (40%), HOMO->L+3 (10%)
13	47464.1150378	210.68548296	30.7692	0.1512	H-6->LUMO (14%), H-1->L+2 (14%), H-1->L+4 (10%)
14	47985.9557534	208.394307105	78.6723	0.0562	H-1->L+2 (43%), H-1->L+4 (15%)
15	48820.7395874	204.830981352	-6.1431	0.0316	H-3->L+1 (36%), H-2->L+1 (37%), HOMO->L+1 (11%)
16	48987.6963542	204.132889363	61.5919	0.1623	H-2->L+2 (25%), H-2->L+4 (14%), H-1->L+4 (16%)
17	49640.1988872	201.449636064	23.772	0.0817	H-2->L+2 (30%), H-2->L+4 (11%)
18	50563.7037084	197.770322713	30.4638	0.0068	H-9->LUMO (76%)
19	51062.9609	195.836665633	1.9921	0.0023	H-3->L+2 (56%)
20	52364.7397484	190.96819822	65.7767	0.028	H-12->LUMO (67%)
21	52825.2823274	189.303294927	-48.2006	0.2499	
22	53072.8945371	188.420098192	-60.0516	0.1431	HOMO->L+6 (42%)
23	53633.4498652	186.450806822	-1.1516	0.0172	H-4->L+1 (25%), HOMO->L+7 (14%)
24	53647.1612905	186.403152738	-14.4931	0.0065	H-4->L+1 (49%)
25	53832.6688092	185.760806982	-24.6588	0.0343	H-3->L+4 (10%), H-1->L+6 (26%), HOMO->L+7 (14%)

26	54173.0347782	184.593682834	-3.5125	0.0246	HOMO->L+3 (17%), HOMO->L+10 (12%)
27	54325.4735653	184.075707835	83.829	0.1451	H-7->L+1 (45%), HOMO->L+7 (11%)
28	54398.8700183	183.827347822	-15.6984	0.0607	H-7->L+1 (18%), HOMO->L+5 (18%)
29	54773.9178278	182.568645745	1.7273	0.0076	H-1->L+5 (18%), H-1->L+7 (25%), H-1->L+8 (25%)
30	55295.7585434	180.845697092	-2.6118	0.0143	H-2->L+6 (16%), H-1->L+3 (14%), H-1->L+5 (15%)
31	55653.0621554	179.684632124	-4.1047	0.0199	
32	55861.1531981	179.015280342	9.8912	0.0321	H-2->L+7 (23%)
33	56066.0180231	178.361159801	-9.442	0.0054	H-2->L+6 (19%)
34	56369.2824884	177.401583958	93.4265	0.1023	
35	56501.5574147	176.98627184	27.179	0.0272	H-2->L+7 (14%), HOMO->L+16 (14%)
36	56863.7003534	175.859114652	-40.8562	0.0343	H-8->L+1 (27%)
37	57283.9152109	174.569073416	-72.536	0.0306	H-8->L+1 (14%), H-4->L+2 (20%)
38	57363.7640993	174.326077743	23.6494	0.09	H-2->L+5 (11%)
39	57634.7663875	173.506385586	-9.4695	0.0395	HOMO->L+12 (29%)
40	57802.5297087	173.00280888	-12.1279	0.013	H-1->L+12 (11%), H-1->L+13 (16%)
41	58050.9484728	172.262473966	14.5444	0.0244	H-10->L+1 (17%)
42	58113.8597183	172.075990968	-14.6578	0.0083	H-13->LUMO (11%), H-5->LUMO (23%)
43	58325.1769787	171.452544476	3.3835	0.0014	H-3->L+6 (19%)
44	58400.1865406	171.232329764	-24.5396	0.0272	H-10->L+1 (32%)
45	58528.4286948	170.857141102	-25.3198	0.0263	H-2->L+4 (11%), H-1->L+10 (11%)
46	58613.1169099	170.610275092	-24.4605	0.0498	
47	58715.5493223	170.312636353	4.158	0.001	H-2->L+10 (16%), H-1->L+10 (16%)
48	58948.6435523	169.63918756	8.7164	0.0155	HOMO->L+11 (11%)
49	59068.8201622	169.294053488	-19.5309	0.0133	H-2->L+3 (10%)
50	59264.0063341	168.736483046	11.1768	0.0298	

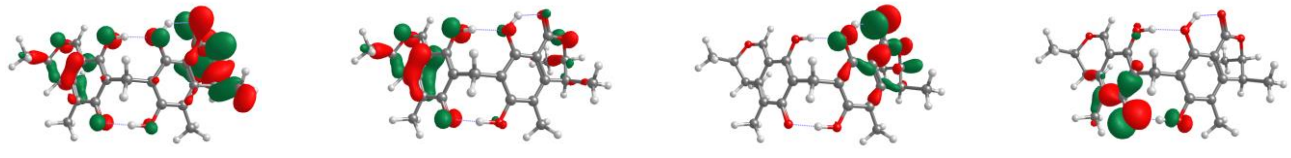

110 **111** **112** **113**

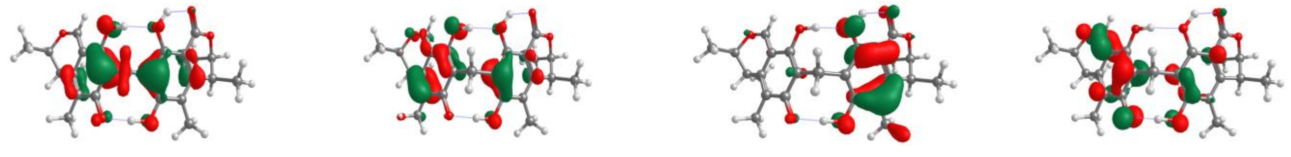

114 **115** **116** **117 (HOMO)**

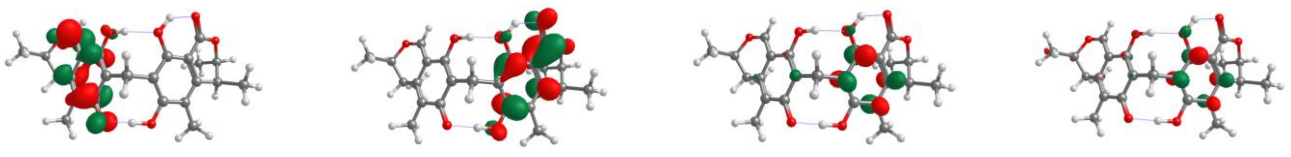

118 (LUMO) **119** **121** **122**

Figure S41. Key molecular orbitals involved in important transitions regarding the ECD spectrum of dominant conformer of **1**.

Table S3. Cartesian coordinate of dominant conformer of **2** (B3LYP/6-31+g(d))

Standard orientation					
Center Number	Atomic Number	Atomic Type	Coordinates (Angstroms)		
			X	Y	Z
1	6	0	-1.17581	0.641875	-0.85339
2	6	0	-1.87999	-0.56877	-0.82636
3	6	0	-3.12301	-0.69514	-0.16326
4	6	0	-3.65416	0.416803	0.538554
5	6	0	-2.95403	1.61582	0.583058
6	6	0	-1.7042	1.708375	-0.09657
7	6	0	-3.82365	-1.97636	-0.21024
8	8	0	-5.0722	-2.0656	0.283662
9	6	0	-5.81642	-0.8436	0.548717
10	6	0	-4.95061	0.180335	1.294311
11	6	0	-6.42998	-0.33053	-0.75239
12	6	0	-4.67678	-0.26262	2.746509
13	8	0	-1.07882	2.886715	0.029355
14	6	0	-3.40625	2.840831	1.343156
15	6	0	0.088424	0.72447	-1.71628
16	6	0	1.398748	0.510182	-0.95474
17	6	0	2.069873	1.615902	-0.29527
18	6	0	3.450446	1.455511	0.209346
19	6	0	4.02186	0.220037	0.214529
20	6	0	3.280213	-0.92657	-0.27792
21	6	0	1.973725	-0.73799	-0.88456
22	8	0	1.521715	2.740212	-0.16005
23	8	0	-1.34341	-1.63986	-1.48044
24	6	0	0.112098	1.934076	-2.68313
25	6	0	3.810198	-2.1826	-0.17723
26	6	0	5.457771	-0.07552	0.617555
27	6	0	5.553037	-1.45955	1.277952
28	8	0	4.952784	-2.4915	0.432849
29	6	0	6.402232	0.039553	-0.59761
30	6	0	4.919917	-1.54173	2.664628

31	6	0	4.104982	2.722647	0.692264
32	8	0	1.411332	-1.86961	-1.35997
33	8	0	-3.3258	-3.00921	-0.67642
34	1	0	-6.61698	-1.1848	1.212275
35	1	0	-5.533	1.107654	1.324279
36	1	0	-6.99077	-1.13124	-1.24374
37	1	0	-7.1188	0.495271	-0.54172
38	1	0	-5.6622	0.032038	-1.44299
39	1	0	-4.14296	-1.21822	2.770121
40	1	0	-4.06507	0.47815	3.270375
41	1	0	-5.6167	-0.38367	3.298542
42	1	0	-0.09212	2.818046	-0.1513
43	1	0	-4.37097	2.698996	1.834177
44	1	0	-2.67175	3.115394	2.109003
45	1	0	-3.48629	3.707026	0.676453
46	1	0	0.011477	-0.13038	-2.39187
47	1	0	-1.961	-2.41467	-1.30198
48	1	0	-0.79782	1.922234	-3.29283
49	1	0	0.180606	2.901383	-2.18935
50	1	0	0.971623	1.83934	-3.35599
51	1	0	3.319789	-3.0517	-0.6012
52	1	0	5.787235	0.644458	1.373965
53	1	0	6.601713	-1.76512	1.333369
54	1	0	6.132258	-0.67822	-1.37907
55	1	0	7.440216	-0.15354	-0.30087
56	1	0	6.350807	1.042778	-1.03107
57	1	0	4.991952	-2.56131	3.054529
58	1	0	5.445904	-0.87059	3.352427
59	1	0	3.865861	-1.24905	2.640391
60	1	0	4.170444	3.456565	-0.11966
61	1	0	3.494609	3.191733	1.471262
62	1	0	5.110727	2.557147	1.08486
63	1	0	0.434461	-1.77744	-1.46413

Table S4. Key transitions and their related rotatory and oscillator strengths of dominant conformer of **2** at the B3LYP/6-31+g(d) level.

HOMO is 121					
**No.**	**Energy (cm-1)**	**Wavelength (nm)**	**R(length)**	**Osc. Strength**	**Major contribs**
1	31041.8603089	322.145641417	15.0025	0.0241	H-3->LUMO (28%), H-2->LUMO (49%), HOMO->LUMO (16%)

2	32347.6719295	309.141258196	-85.6973	0.5604	H-2->LUMO (21%), HOMO->LUMO (74%)
3	35067.3734641	285.16535492	9.1473	0.0059	H-4->LUMO (82%)
4	35827.1477362	279.11794915	-19.6469	0.1564	H-1->L+1 (79%)
5	38312.9484864	261.008363884	93.9138	0.235	H-3->L+1 (11%), H-2->L+1 (29%), HOMO->L+1 (31%)
6	39578.4323855	252.662862	1.402	0.021	H-1->LUMO (69%)
7	40317.2362424	248.032874572	8.3953	0.0073	H-3->LUMO (57%), H-2->LUMO (16%), H-1->LUMO (13%)
8	44516.9651542	224.633461993	-21.1895	0.0999	H-5->L+1 (12%), H-3->L+1 (20%), HOMO->L+1 (32%)
9	45404.9815806	220.240151012	-106.7086	0.1361	H-5->L+1 (54%), HOMO->L+3 (10%)
10	45768.737628	218.489749079	-53.7928	0.2105	H-5->L+1 (14%), H-3->L+1 (11%), HOMO->L+1 (16%), HOMO->L+3 (17%), HOMO->L+4 (11%)
11	46898.7203831	213.225433835	-41.6616	0.0182	H-6->LUMO (43%)
12	47239.8929065	211.685492594	44.51	0.1782	H-6->LUMO (29%), H-1->L+3 (17%)
13	47292.3189444	211.450828025	-2.1152	0.0011	HOMO->L+2 (63%)
14	47884.3298953	208.836586455	52.3126	0.0255	H-1->L+2 (56%), H-1->L+4 (10%)
15	48611.8419903	205.7111928	64.6593	0.19	H-2->L+3 (17%), H-2->L+4 (12%), H-1->L+3 (10%), H-1->L+4 (13%)
16	48957.8538403	204.257319625	41.046	0.021	H-3->L+1 (39%), H-2->L+1 (43%)
17	49414.3636471	202.370308184	8.9112	0.0053	H-2->L+2 (57%), HOMO->L+4 (10%)
18	50523.375987	197.928182839	38.8864	0.0094	H-10->LUMO (50%), H-9->LUMO (22%), H-6->LUMO (10%)
19	51625.9358914	193.701088945	1.3218	0.003	H-3->L+2 (46%)
20	52092.9309058	191.964626027	87.3992	0.0428	H-12->LUMO (63%)
21	52723.6564693	189.668180655	-107.0515	0.3619	H-3->L+3 (14%), H-2->L+10 (11%)
22	53079.3469725	188.397193454	-23.3197	0.0264	HOMO->L+6 (44%), HOMO->L+9 (11%)
23	53180.1662761	188.040028835	-15.243	0.0027	H-4->L+1 (72%)
24	53364.8672404	187.389204117	7.5617	0.0034	H-1->L+5 (28%), H-1->L+6 (16%)
25	53723.7839613	186.137298281	-15.0931	0.0725	HOMO->L+7 (16%)
26	54143.9988188	184.692675424	31.0952	0.07	H-7->L+1 (25%), HOMO->L+4 (13%), HOMO->L+5 (15%)
27	54222.2345984	184.426187413	40.402	0.091	H-7->L+1 (25%), HOMO->L+5 (12%)
28	54390.804474	183.85460735	41.2135	0.0375	H-3->L+3 (15%), H-3->L+4 (11%), HOMO->L+7 (20%)
29	54569.8595573	183.251342062	9.1442	0.0048	H-1->L+4 (10%), H-1->L+7 (37%), H-1->L+8 (18%)
30	54994.9137414	181.834997451	-11.5744	0.0736	H-2->L+5 (11%), H-2->L+6 (10%)
31	55440.9383406	180.372127516	3.6031	0.0022	H-1->L+6 (12%)
32	55705.4881933	179.515525747	-25.5729	0.0203	H-2->L+7 (29%)
33	55795.0157349	179.227478804	-39.7834	0.0108	H-1->L+3 (10%), H-1->L+6 (15%)
34	56147.4800204	178.102383159	43.5683	0.0726	H-8->L+1 (12%), H-4->L+3 (10%),

					H-4->L+10 (11%)
35	56200.7126127	177.933686872	15.1032	0.0337	H-8->L+1 (34%)
36	56527.3671565	176.905461957	-43.3453	0.0428	
37	57066.145515	175.235245166	-12.7065	0.0635	
38	57283.1086564	174.571531374	15.9084	0.0052	HOMO->L+13 (29%)
39	57381.5082968	174.272170545	19.8864	0.0347	H-5->LUMO (31%)
40	57610.5697546	173.579258851	28.0083	0.0131	H-1->L+13 (13%), H-1->L+14 (12%)
41	57668.6416735	173.404465751	-44.3229	0.0336	H-4->L+2 (13%)
42	57760.5888784	173.128428816	-25.3099	0.0841	H-4->L+2 (12%)
43	58013.8469691	172.372640713	-11.4515	0.0027	H-2->L+10 (14%)
44	58068.6926703	172.209835286	-0.3288	0.0037	
45	58209.0331409	171.794641835	5.1721	0.02	
46	58286.4623661	171.566425445	-18.9277	0.0241	H-9->L+1 (18%)
47	58511.4910518	170.906600058	-6.4169	0.5604	H-14->LUMO (11%), H-13->LUMO (10%)
48	58620.3758997	170.589148338	-6.3	0.0059	H-2->L+8 (13%)
49	58792.1719931	170.090671275	-18.4286	0.1564	HOMO->L+11 (17%)
50	59028.4924408	169.409713623	-1.2677	0.235	H-2->L+4 (16%)

Figure S42. Key molecular orbitals involved in important transitions regarding the ECD spectrum of dominant conformer of **2**.

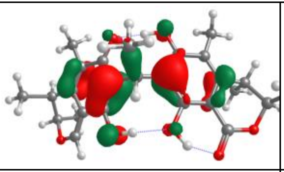	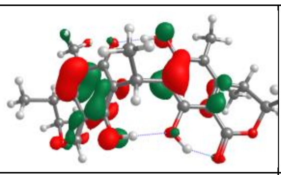	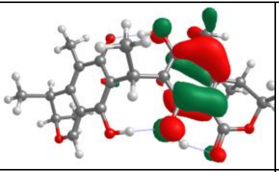	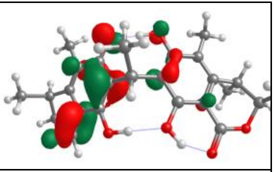
**118**	**119**	**120**	**121 (HOMO)**
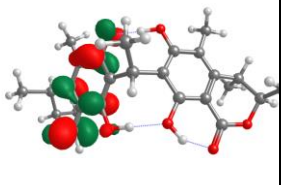	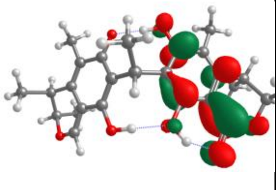	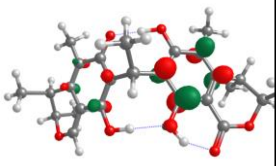	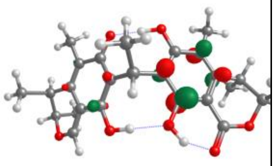
**122 (LUMO)**	**123**	**125**	**126**

Table S5. Cartesian coordinate of dominant conformer of **3** (B3LYP/6-31+g(d))

Standard orientation					
Center Number	Atomic Number	Atomic Type	Coordinates (Angstroms)		
			X	Y	Z
1	6	0	-5.04882	0.63821	1.004304
2	6	0	-5.91255	-0.61482	0.749594
3	8	0	-5.1577	-1.78244	1.070773
4	6	0	-4.0294	-1.99235	0.231877

5	6	0	-3.24869	-0.73387	-0.08339
6	6	0	-3.72738	0.537489	0.250352
7	6	0	-2.02244	-0.88045	-0.75991
8	6	0	-1.26115	0.243065	-1.12062
9	6	0	-1.7628	1.495036	-0.75379
10	6	0	-2.97416	1.68665	-0.08074
11	6	0	1.300997	-0.06724	-1.10257
12	6	0	2.069584	0.979982	-0.66625
13	6	0	3.345694	0.744732	-0.01479
14	6	0	3.786211	-0.61599	0.243415
15	6	0	3.016122	-1.67306	-0.1364
16	6	0	1.726519	-1.42751	-0.81658
17	6	0	4.121577	1.802908	0.36583
18	8	0	5.356607	1.716639	0.859201
19	6	0	6.021833	0.419868	0.737304
20	6	0	5.057751	-0.72886	1.070743
21	8	0	-1.65436	-2.14638	-1.06799
22	6	0	-6.5278	-0.66793	-0.65341
23	6	0	-4.81741	0.82476	2.517668
24	6	0	-3.40912	3.101236	0.243606
25	8	0	-0.98389	2.591962	-1.12747
26	6	0	0.029598	0.120967	-1.91265
27	8	0	1.017518	-2.41189	-1.1372
28	6	0	4.729947	-0.76942	2.578438
29	6	0	6.662598	0.328498	-0.64545
30	6	0	3.336344	-3.1214	0.121167
31	8	0	1.750578	2.282604	-0.84023
32	1	0	-5.61462	1.501587	0.629472
33	1	0	-6.72999	-0.62286	1.48089
34	1	0	-4.33365	-2.48249	-0.70751
35	1	0	-3.3927	-2.71063	0.757764
36	1	0	3.778384	2.828546	0.286848
37	1	0	6.803516	0.471401	1.500554
38	1	0	5.58574	-1.65323	0.812499
39	1	0	-0.67122	-2.22274	-1.20595
40	1	0	-5.77093	-0.58478	-1.44005
41	1	0	-7.06764	-1.61093	-0.78947
42	1	0	-7.23915	0.155881	-0.78506
43	1	0	-5.76867	0.991252	3.038619
44	1	0	-4.35276	-0.07372	2.934677

45	1	0	-4.16111	1.676013	2.727236
46	1	0	-3.51115	3.720781	-0.66058
47	1	0	-4.37629	3.131228	0.745966
48	1	0	-2.70069	3.611411	0.915179
49	1	0	-1.38559	3.402888	-0.78154
50	1	0	-0.0559	-0.73768	-2.58635
51	1	0	0.135713	1.003895	-2.55178
52	1	0	5.646164	-0.87963	3.17106
53	1	0	4.07085	-1.61195	2.807216
54	1	0	4.225658	0.14821	2.898674
55	1	0	7.307059	1.194745	-0.82222
56	1	0	7.275966	-0.57691	-0.70984
57	1	0	5.907525	0.286385	-1.43613
58	1	0	2.60214	-3.56279	0.805872
59	1	0	3.261805	-3.69673	-0.80758
60	1	0	4.331762	-3.26733	0.54705
61	1	0	0.791329	2.383144	-1.03868

Table S6. Key transitions and their related rotatory and oscillator strengths of dominant conformer of **3** at the B3LYP/6-31+g(d) level.

HOMO is 114					
**No.**	**Energy (cm-1)**	**Wavelength (nm)**	**R(length)**	**Osc. Strength**	**Major contribs**
1	31074.9290405	321.802826548	8.9055	0.0285	H-3->LUMO (57%), H-2->LUMO (20%), H-1->LUMO (16%)
2	32418.6487192	308.464430045	1.8901	0.5886	H-2->LUMO (27%), H-1->LUMO (47%), HOMO->LUMO (18%)
3	34775.4007608	287.559590436	-30.5325	0.0056	H-5->LUMO (73%)
4	35751.3316199	279.709861057	6.153	0.0168	H-1->LUMO (17%), HOMO->LUMO (74%)
5	39088.0472926	255.83268269	7.2001	0.0029	H-5->LUMO (12%), H-3->LUMO (27%), H-2->LUMO (37%), H-1->LUMO (16%)
6	40739.0642088	245.464646629	-4.9812	0.0453	HOMO->L+3 (39%)
7	43651.5322519	229.087032783	5.9182	0.0017	HOMO->L+1 (69%)
8	45350.1358794	220.506505793	7.1155	0.1264	H-1->L+1 (20%), H-1->L+3 (15%)
9	46349.4568169	215.752258748	30.9471	0.071	H-1->L+1 (32%), HOMO->L+9 (16%)
10	46764.8323478	213.835899712	61.6484	0.1189	H-2->L+1 (10%), H-1->L+1 (13%), H-1->L+3 (11%)
11	47575.419549	210.192576225	44.1847	0.0209	H-8->LUMO (23%), H-7->LUMO (62%)
12	48146.4600847	207.699589594	-6.395	0.0128	H-2->L+1 (46%), H-1->L+2 (13%)
13	49029.6371845	203.958270431	-0.6063	0.0469	HOMO->L+4 (41%), HOMO->L+5 (18%)
14	49618.4219177	201.538050053	-285.9488	0.5643	H-2->L+3 (17%), H-1->L+3 (10%), HOMO->L+9 (17%)

15	50064.4465169	199.742545773	18.5427	0.0108	H-10->LUMO (11%), H-9->LUMO (69%)
16	50344.3209038	198.632136068	-47.4457	0.084	H-3->L+1 (47%), H-2->L+2 (10%)
17	50911.3286673	196.419937601	-6.4892	0.1372	HOMO->L+5 (15%), HOMO->L+7 (30%)
18	51396.8744336	194.564360386	88.2714	0.1946	H-1->L+9 (11%), HOMO->L+3 (26%)
19	51756.5977089	193.212081989	21.2041	0.0111	HOMO->L+2 (25%), HOMO->L+5 (15%), HOMO->L+7 (10%)
20	51831.6072708	192.932469714	3.4017	0.0021	H-4->LUMO (91%)
21	52492.9819026	190.501656365	-10.0275	0.0215	H-1->L+5 (35%)
22	52790.6004869	189.427661511	-39.4592	0.2263	H-11->LUMO (16%), H-1->L+4 (13%)
23	52915.6164234	188.980128663	111.0134	0.09	H-12->LUMO (15%), H-11->LUMO (42%)
24	53329.3788455	187.513903527	35.9615	0.0554	H-1->L+2 (15%)
25	53483.4307414	186.973794713	6.8029	0.0121	H-1->L+7 (15%), HOMO->L+11 (12%)
26	53622.1581032	186.49006966	3.5787	0.0011	H-1->L+2 (19%), H-1->L+6 (13%)
27	53936.7143305	185.402468877	-7.4183	0.0636	H-2->L+4 (11%), H-1->L+3 (15%)
28	54110.9300872	184.805546382	41.5273	0.0416	H-2->L+3 (10%), H-2->L+4 (17%)
29	54627.9314762	183.056537741	-74.8624	0.1666	H-1->L+10 (11%), HOMO->L+6 (22%)
30	54839.2487366	182.351148683	13.4549	0.0204	H-2->L+5 (14%), H-2->L+6 (13%)
31	55270.7553561	180.927507424	24.7706	0.0382	H-2->L+2 (16%)
32	55393.3516293	180.52707962	9.643	0.0305	
33	55620.7999783	179.788856038	-15.1548	0.0158	H-4->L+4 (16%), HOMO->L+13 (14%)
34	55770.8191021	179.305238134	3.82	0.0149	HOMO->L+11 (16%)
35	56177.3225343	178.007771622	-8.9393	0.0272	H-4->L+3 (15%)
36	56306.3712429	177.599795179	-10.3009	0.0692	H-3->L+2 (12%)
37	56395.8987845	177.317858488	27.3873	0.0178	H-5->L+1 (10%)
38	56612.8619259	176.638305498	-16.9143	0.0141	HOMO->L+12 (12%), HOMO->L+14 (12%)
39	56686.258379	176.409597069	-19.6675	0.0322	H-4->L+3 (26%)
40	56803.2087712	176.046392736	-24.9029	0.0063	H-4->L+4 (11%), H-1->L+11 (10%)
41	57018.5588037	175.381493496	24.7012	0.0317	
42	57137.9288592	175.01509417	7.8765	0.0225	H-5->L+1 (14%)
43	57283.9152109	174.569073416	19.9078	0.0074	H-3->L+9 (15%), H-1->L+9 (12%)
44	57555.7240534	173.744665096	27.773	0.0424	HOMO->L+16 (38%)
45	57605.730428	173.59384085	2.8985	0.0034	H-3->L+5 (11%), H-1->L+12 (21%)
46	57953.3553869	172.552562888	8.8962	0.0498	
47	58147.7350043	171.975744184	0.1536	0.001	
48	58278.3968218	171.590169691	25.7624	0.0155	H-8->LUMO (10%)
49	58511.4910518	170.906600058	-26.8049	0.0133	H-2->L+12 (19%)
50	58616.3431276	170.600884778	-3.5314	0.0298	

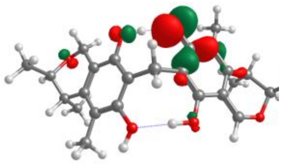

109

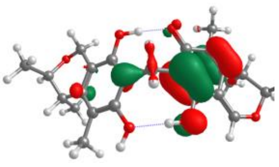

111

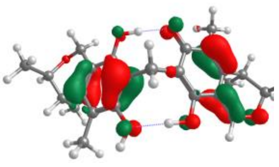

112

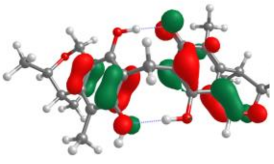

113

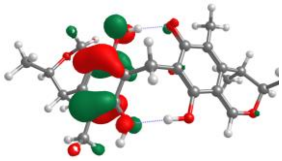

114 (HOMO)

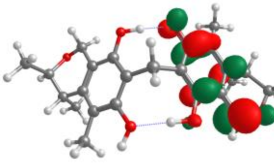

115 (LUMO)

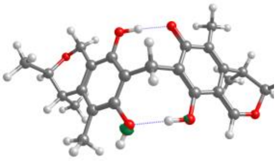

116

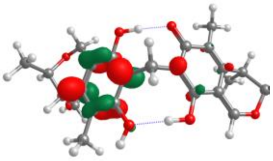

118

Figure S43. Key molecular orbitals involved in important transitions regarding the ECD spectrum of dominant conformer of **3**.

Table S7. Cartesian coordinate of dominant conformer of **4** (B3LYP/6-31+g(d))

Standard orientation					
Center Number	Atomic Number	Atomic Type	Coordinates (Angstroms)		
			X	Y	Z
1	6	0	1.167545	0.302173	0.4098
2	6	0	1.874225	0.171406	-0.76447
3	6	0	3.080214	-0.64333	-0.80675
4	6	0	3.561019	-1.30331	0.393927
5	6	0	2.831803	-1.2491	1.542773
6	6	0	1.557054	-0.50144	1.555511
7	6	0	3.755237	-0.78552	-1.9859
8	8	0	4.816773	-1.56778	-2.18066
9	6	0	5.119891	-2.54303	-1.1349
10	6	0	4.937591	-1.93591	0.265082
11	6	0	4.289694	-3.79878	-1.38952
12	6	0	6.043358	-0.9081	0.585241
13	8	0	0.846512	-0.55588	2.595904
14	6	0	3.212414	-1.89108	2.850617
15	6	0	-0.04018	1.214658	0.608348
16	6	0	-1.36664	0.576818	0.202688
17	6	0	-1.80778	0.595999	-1.17589
18	6	0	-3.19685	0.216277	-1.51402
19	6	0	-3.99265	-0.33043	-0.55377
20	6	0	-3.47657	-0.54062	0.787236
21	6	0	-2.15417	-0.05154	1.142812
22	8	0	-1.05229	0.960274	-2.11795
23	8	0	1.536235	0.755277	-1.92195
24	6	0	-4.23031	-1.19511	1.719591
25	6	0	-5.46298	-0.67487	-0.72906
26	6	0	-5.82238	-1.91769	0.100845
27	8	0	-5.41662	-1.76023	1.496593

28	6	0	-6.35547	0.529063	-0.36101
29	6	0	-5.23245	-3.22187	-0.43014
30	6	0	-3.60367	0.479455	-2.9398
31	8	0	-1.78912	-0.25747	2.418311
32	6	0	0.113902	2.668232	0.086792
33	6	0	1.334225	3.372328	0.683257
34	6	0	1.39969	4.838804	0.320238
35	8	0	0.55563	5.473802	-0.27272
36	8	0	2.5561	5.406965	0.757027
37	1	0	3.462303	-0.25172	-2.88316
38	1	0	6.178552	-2.76272	-1.30056
39	1	0	5.032652	-2.76789	0.970867
40	1	0	3.222451	-3.60862	-1.24063
41	1	0	4.443059	-4.15333	-2.41308
42	1	0	4.595923	-4.59244	-0.69912
43	1	0	5.920985	-0.51475	1.598825
44	1	0	6.011054	-0.06248	-0.10949
45	1	0	7.035173	-1.37086	0.513796
46	1	0	3.360971	-1.13085	3.627167
47	1	0	2.397991	-2.52926	3.208993
48	1	0	4.124304	-2.48882	2.781302
49	1	0	-0.10078	1.33195	1.694468
50	1	0	0.538849	0.926915	-1.95971
51	1	0	-3.90349	-1.30685	2.747486
52	1	0	-5.66189	-0.93943	-1.77293
53	1	0	-6.91045	-2.00747	0.168252
54	1	0	-6.21677	0.819621	0.685411
55	1	0	-7.41452	0.286209	-0.51044
56	1	0	-6.1111	1.394136	-0.98461
57	1	0	-5.62882	-3.42789	-1.43051
58	1	0	-4.14167	-3.16806	-0.49864
59	1	0	-5.50172	-4.05469	0.226231
60	1	0	-3.54268	1.550398	-3.16773
61	1	0	-2.9097	-0.01331	-3.62886
62	1	0	-4.61868	0.141205	-3.16006
63	1	0	-0.78695	-0.27561	2.513718
64	1	0	-0.78869	3.217298	0.377026
65	1	0	0.159254	2.724654	-1.00001
66	1	0	2.270577	2.903123	0.359894
67	1	0	1.337414	3.306313	1.780851

68	1	0	2.505887	6.344486	0.491567

Table S8. Key transitions and their related rotatory and oscillator strengths of dominant conformer of **4** at the B3LYP/6-31+g(d) level.

HOMO is 132					
**No.**	**Energy (cm-1)**	**Wavelength (nm)**	**R(length)**	**Osc. Strength**	**Major contribs**
1	29512.63	338.8379	8.9055	0.001	H-3->L+1 (16%), H-2->LUMO (25%), HOMO->LUMO (56%)
2	30572.45	327.0919	1.8901	0.056	H-3->LUMO (24%), H-2->L+1 (38%), HOMO->L+1 (29%)
3	32419.46	308.4568	-30.5325	0.9805	H-1->LUMO (44%), HOMO->L+1 (44%)
4	32570.28	307.0284	6.153	0.1419	H-2->LUMO (30%), H-1->L+1 (41%), HOMO->LUMO (23%)
5	35275.46	283.4832	7.2001	0.0043	H-5->LUMO (10%), H-5->L+1 (27%), H-4->LUMO (28%), H-4->L+1 (10%)
6	35423.87	282.2955	-4.9812	0.0015	H-5->LUMO (35%), H-4->LUMO (15%), H-4->L+1 (25%)
7	38010.49	263.0853	5.9182	0.0091	H-3->LUMO (36%), H-1->LUMO (20%), HOMO->L+1 (18%)
8	38564.59	259.3052	7.1155	0.0041	H-3->L+1 (32%), H-1->L+1 (28%), HOMO->LUMO (13%)
9	41403.67	241.5245	30.9471	0.0047	H-3->LUMO (12%), H-3->L+1 (13%), H-2->LUMO (24%), H-2->L+1 (24%), H-1->LUMO (12%), H-1->L+1 (11%)
10	41741.61	239.5691	61.6484	0.0049	H-3->LUMO (12%), H-3->L+1 (20%), H-2->LUMO (17%), H-2->L+1 (27%), H-1->L+1 (12%)
11	45379.17	220.3654	44.1847	0.3215	HOMO->L+5 (46%)
12	45842.94	218.1361	-6.395	0.0063	H-8->L+1 (14%), H-5->LUMO (18%), H-5->L+1 (10%), H-4->L+1 (15%)
13	45899.4	217.8678	-0.6063	0.035	H-7->LUMO (11%), H-7->L+1 (14%), H-5->L+1 (20%), H-4->LUMO (11%)
14	46618.04	214.5092	-285.9488	0.0025	HOMO->L+2 (65%)
15	46752.73	213.8912	18.5427	0.0151	H-8->L+1 (10%), H-7->LUMO (19%), H-5->LUMO (15%), H-4->L+1 (24%)
16	47060.03	212.4945	-47.4457	0.0238	H-1->L+5 (27%), HOMO->L+12 (10%), HOMO->L+13 (10%)

17	47350.39	211.1915	-6.4892	0.0162	H-8->LUMO (28%), H-7->L+1 (14%), H-5->L+1 (14%), H-4->LUMO (13%)
18	48267.44	207.179	88.2714	0.001	H-1->L+2 (52%), HOMO->L+3 (15%)
19	48431.17	206.4786	21.2041	0.0003	H-6->L+6 (16%), H-6->L+7 (10%), H-6->L+8 (42%), H-6->L+9 (16%)
20	49514.38	201.9615	3.4017	0.0138	H-2->L+2 (49%), H-1->L+3 (21%)
21	49832.16	200.6736	-10.0275	0.3562	H-2->L+5 (27%), H-1->L+12 (10%)
22	50660.49	197.3925	-39.4592	0.0137	H-11->L+1 (10%), H-10->L+1 (17%), H-9->LUMO (22%)
23	50750.02	197.0443	111.0134	0.0027	H-3->L+2 (20%), HOMO->L+3 (14%)
24	50871	196.5756	35.9615	0.027	H-11->LUMO (12%), H-11->L+1 (10%), H-10->LUMO (21%), H-9->L+1 (18%)
25	52005.82	192.2862	6.8029	0.009	HOMO->L+4 (16%), HOMO->L+6 (43%)
26	52255.05	191.3691	3.5787	0.0336	
27	52466.37	190.5983	-7.4183	0.008	
28	52495.4	190.4929	41.5273	0.0228	
29	52675.26	189.8424	-74.8624	0.0629	H-12->LUMO (20%), H-12->L+1 (20%)
30	52945.46	188.8736	13.4549	0.0017	H-1->L+6 (23%), HOMO->L+7 (15%)
31	53280.18	187.6871	24.7706	0.0072	H-1->L+5 (15%), HOMO->L+3 (11%)
32	54014.14	185.1367	9.643	0.0001	H-1->L+4 (22%)
33	54169	184.6074	-15.1548	0.0023	H-1->L+6 (16%)
34	54390.8	183.8546	3.82	0.0024	H-2->L+6 (14%), HOMO->L+8 (13%)
35	54670.68	182.9134	-8.9393	0.0711	H-3->L+5 (16%)
36	54790.86	182.5122	-10.3009	0.0272	H-2->L+4 (15%), H-1->L+7 (11%)
37	55132.03	181.3828	27.3873	0.0112	H-2->L+6 (11%)
38	55432.87	180.3984	-16.9143	0.033	
39	55687.74	179.5727	-19.6675	0.0242	
40	55943.42	178.752	-24.9029	0.0577	
41	56058.76	178.3843	24.7012	0.0044	H-14->LUMO (13%), HOMO->L+18 (10%)
42	56291.05	177.6481	7.8765	0.0063	HOMO->L+14 (15%)
43	56406.38	177.2849	19.9078	0.0311	
44	56647.54	176.5302	27.773	0.1174	H-5->L+5 (12%)
45	56708.04	176.3419	2.8985	0.0752	
46	56808.05	176.0314	8.8962	0.1875	
47	57112.93	175.0917	0.1536	0.0185	H-4->L+5 (19%)
48	58948.6435523	169.63918756	25.7624	0.003	
49	59068.8201622	169.294053488	-26.8049	0.0477	
50	59264.0063341	168.736483046	-3.5314	0.0427	H-5->L+2 (12%)

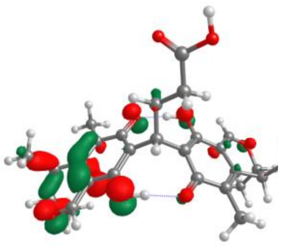

124

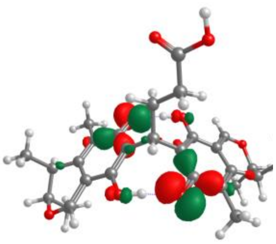

127

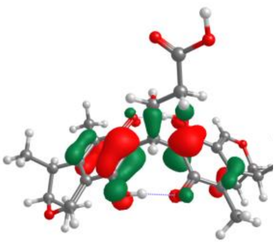

129

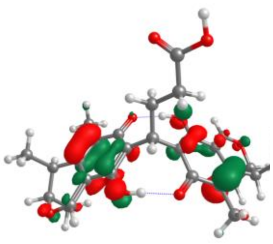

130

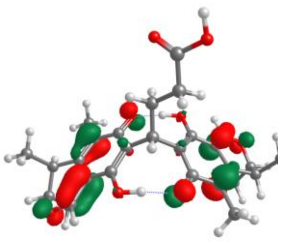

131

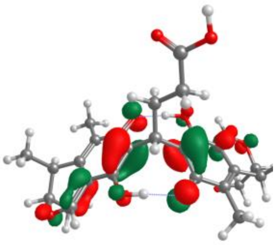

132 (HOMO)

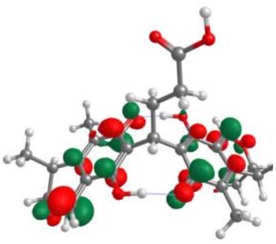

133 (LUMO)

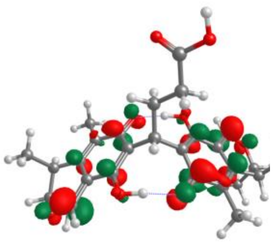

134

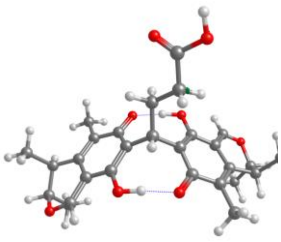

135

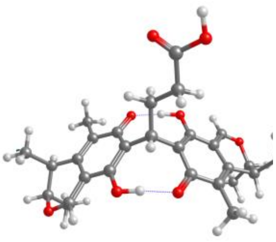

136

Figure S44. Key molecular orbitals involved in important transitions regarding the ECD spectrum of dominant conformer of **4**.
